# 3,4-Unsubstituted 2-*tert*-Butyl-pyrrolidine-1-oxyls with Hydrophilic Functional Groups in the Side Chains

**DOI:** 10.3390/molecules27061922

**Published:** 2022-03-16

**Authors:** Andrey I. Taratayko, Yurii I. Glazachev, Ilia V. Eltsov, Elena I. Chernyak, Igor A. Kirilyuk

**Affiliations:** 1 N.N. Vorozhtsov Institute of Organic Chemistry SB RAS, Academician Lavrentiev Ave. 9, 630090 Novosibirsk, Russia; chernyak@nioch.nsc.ru (E.I.C.); kirilyuk@nioch.nsc.ru (I.A.K.); 2 Voevodsky Institute of Chemical Kinetics and Combustion SB RAS, Institutskaya 3, 630090 Novosibirsk, Russia; glaza@kinetics.nsc.ru; 3 Department of Natural Sciences, Novosibirsk State University, Pirogova Str. 1, 630090 Novosibirsk, Russia; eiv@fen.nsu.ru

**Keywords:** nitroxides, nitrones, organometallic compounds, pyrrolidine, reduction kinetics

## Abstract

Pyrrolidine nitroxides with four bulky alkyl substituents adjacent to N–O group are known for their high resistance to bioreduction. The 3,4-unsubstituted 2-*tert*-butyl-2-ethylpyrrolidine-1-oxyls were prepared from the corresponding 2-*tert*-butyl-1-pyrroline-1-oxides via either the addition of ethinylmagnesium bromide with subsequent hydrogenation or via treatment with ethyllithium. The new nitroxides showed excellent stability to reduction with ascorbate with no evidence for additional large hyperfine couplings in the EPR spectra.

## 1. Introduction

Nitroxides are a broad family of stable free radicals with one unpaired electron localized on the unbonding orbital of the N–O group. These radicals have found broad application in various fields of science and technology [1,2,3]. The broad majority of commonly used nitroxides have a cyclic structure with four methyl substituents adjacent to the N–O group. Recently, nitroxides with methyl groups replaced with bulkier alkyl substituents have attracted much attention. These so-called “sterically shielded” nitroxides demonstrated much higher stability against chemical reduction to diamagnetic compounds with components of biological systems then their tetramethyl analogs did [4,5,6]. The advantage of these reduction-resistant radicals over conventional tetramethyl-substituted nitroxides is especially obvious when they are used for EPR measurements inside living cells [7,8], or in vivo for functional imaging using MRI or EPRI techniques [9,10]. Pyrrolidine nitroxides with four ethyl groups (or three ethyl and one *tert*-butyl group) in the nearest environment of the nitroxide moiety demonstrated the highest resistance to reduction [4,5,11,12,13,14]. However, nitroxides with multiple bulky alkyl substituents are lipophilic or even insoluble in water, therefore their use in biophysics and structural biology is complicated [14]. Moreover, the EPR spectra of these nitroxides may reveal large additional hyperfine couplings (ca. 0.2 mT) with methylene hydrogens of the ethyl groups [11,13,14]. These couplings are typical for 3-substituted or 3,4-disubstituted five-membered rings sterically shielded nitroxides and were shown to result from interaction of ethyl group with a cis-substituent at the neighboring asymmetric center, which prevents averaging of spin density on methylene hydrogens of the ethyl group [15].

The 3,4-unsubstututed sterically shielded pyrrolidine nitroxides with carboxylic groups in the side chains were described by Lampp et al. [12]. The EPR spectra of these nitroxides showed relatively narrow lines (ΔB_pp_ (Gaussian) = 0.18–0.21 mT) with no evidence for large additional splittings on γ-hydrogens. Here, we combined the general approach used by Lampp with the methods suggested in our previous papers [11,13,14] to prepare new water-soluble sterically shielded nitroxides.

## 2. Results and Discussion

**Scheme 1:**
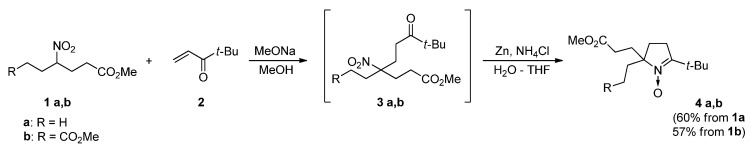
Synthesis of pyrroline *N*-oxides.

Reductive cyclization of aliphatic γ-nitroketones is one of the most widely used methods of synthesis of pyrroline *N*-oxides [12,16,17,18], and reactions of the latter with organometallic reagents are commonly used for the preparation of pyrrolidine nitroxides [12,16]. Bulky substituents and functional groups can be introduced on the first step of this sequence of procedures. Sodium methoxide-catalyzed Michael addition of nitrocompound **1a,b** [19,20] to 4,4-dimethylpent-1-en-3-one 2 [21] afforded γ-nitroketone **3a,b** (Scheme 1), which were treated with Zn/NH_4_Cl without purification. Resulting nitrones **4a,b** were isolated as a colorless oils with 57–60% yields for two steps. 

**Scheme 2:**
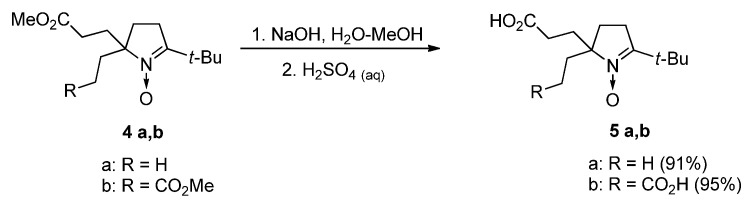
Alkaline hydrolysis of **4a,b**.

The addition of ethylnylmagnesium bromide to pyrroline *N*-oxides can proceed without affecting the carboxylic group, and the ethynyl moiety can be converted into an ethyl group via hydrogenation [13]. Here, we exploited the same approach. Alkaline hydrolysis of ester groups in **4a,b** gave corresponding mono- and dicarboxylic acids **5a,b** with nearly quantitative yields (Scheme 2).

**Scheme 3:**
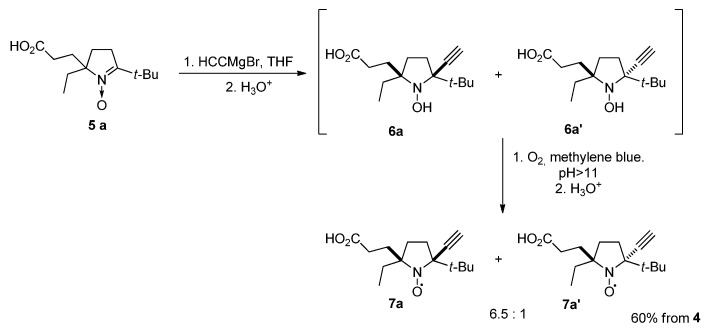
Addition of ethynylmagnesium bromide to nitrone **5a**.

The addition of ethynylmagnesium bromide to **5a** proceeded slowly, presumably due to sterical hindrance. Complete conversion was achieved only on the 7th day of treatment with 15-fold excess (0.5–1 M in THF) of the reagent at room temperature. Subsequent treatment analogous to the literature procedure [13] afforded a mixture of two diastereomeric nitroxides, **7a** and **7a′**, which could not be separated by column chromatography or crystallization (Scheme 3). The ratio of isomers **7a** and **7a′** was 6.5:1 according to ^1^H NMR spectra recorded after reduction of the sample with Zn in the presence of trifluoroacetic acid in CD_3_OD (see [5] for detailed procedure of reduction).

**Scheme 4:**
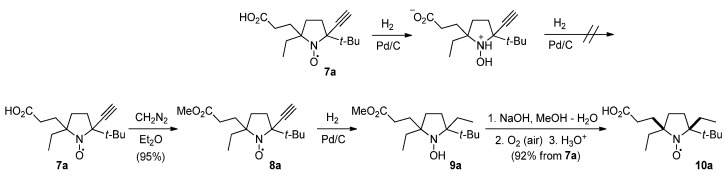
Synthesis of nitroxide **10a**.

Similar to that described for 3-carboxypyrrolidine nitroxides [13], the hydrogenation of a mixture of **7a**,**a′** on Pd/C in methanolic solution gives a crystalline precipitate of poorly soluble zwitterionic 1-hydroxypyrrolidinium carboxylates. As a result, the addition of hydrogen to multiple carbon-carbon bonds occurs slowly, leaving no chance to obtain the desired 2,5-diethyl-substituted nitroxide with a satisfactory yield [13]. To prevent the precipitation of inner salts, mixture **7a,a′** was first treated with diazomethane solution (Scheme 4), and resulting mixture of esters **8a** was subjected to hydrogenation at atmospheric pressure on Pd/C (4%) in methanolic solution. Subsequent treatment with alkali in aqueous methanol in aerobic conditions afforded **10a** in 92% yield. Pure major isomer **10a** was isolated via crystallization from hexane. To determine the relative configuration of the asymmetric centers in **10a**, the nitroxide was reduced to corresponding amine with Zn in CF_3_COOH–CD_3_OD at 65 °C and NMR spectra were recorded. Analysis of the HSQC, HMBC, COSY, and NOESY spectra unambiguously showed the trans-configuration of the ethyl groups in the pyrrolidine ring (see Supplementary Materials SI and Appendix A). Thus, the addition of the ethylnylmagnesium bromide to **5a** predominantly occurs cis to the 2-carboxyethyl group, presumably due to coordination of the organometallic reagent with carboxylate anion. 

**Scheme 5:**
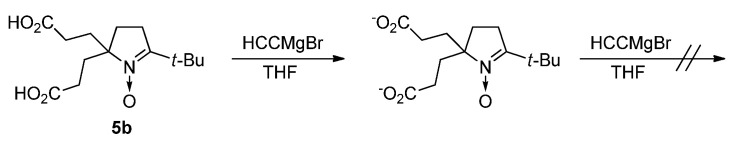
Reaction of nitrone **5b** with ethynylmagnesium bromide.

In contrast to **5a**, no trace of conversion was observed upon treatment of nitrone **5b** with a twenty-fold excess of the ethylnylmagnesium bromide, even with an increase in reaction time of up to 3 months. The explanation is most likely the formation of insoluble magnesium salt (Scheme 5).

**Scheme 6:**
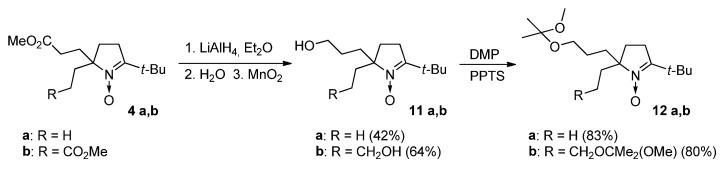
Synthesis of the nitrones **12a,b**.

Organolithium compounds can be successfully used to prepare highly strained nitroxides from cyclic α-*tert*-butylnitrones [14]. To avoid side reactions and increased reagent consumption, esters were reduced to hydroxymethyl groups and the latter were protected. Treatment of nitrones **4a,b** with a threefold excess of lithium aluminum hydride resulted in reduction of both the ester and nitrone groups (Scheme 6). To regenerate the nitrone groups, the resulting *N*-hydroxyamines were oxidized with MnO_2_. The hydroxy groups were protected via treatment with dimethoxypropane in the presence of pyridinium tosylate. Pure nitrones **12a,b** were isolated using column chromatography as colorless oils. 

**Scheme 7:**
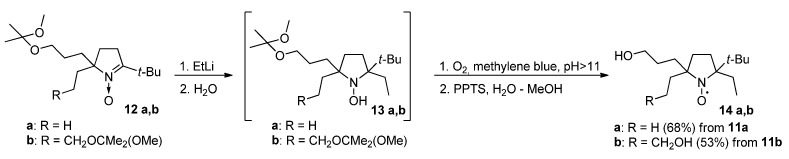
Synthesis of nitroxides **14a,b**.

Nitrones **12a,b** readily reacted with ethyllithium, the resulting hydroxylamines **13a,b** were oxidized to nitroxides by air oxygen in presence of methylene blue without isolation, and the protecting groups were removed to give **14a,b** (Scheme 7).

HPLC analysis and ^1^H NMR spectra after reduction of the sample with Zn in the presence of trifluoroacetic acid in CD_3_OD showed that **14a** is a mixture of diastereomers in equal ratio. Thus, unlike the literature examples [14], addition of ethyllithium to **12a** does not exhibit any stereoselectivity. The nitroxide **14b** is a racemate. 

**Figure 1:**
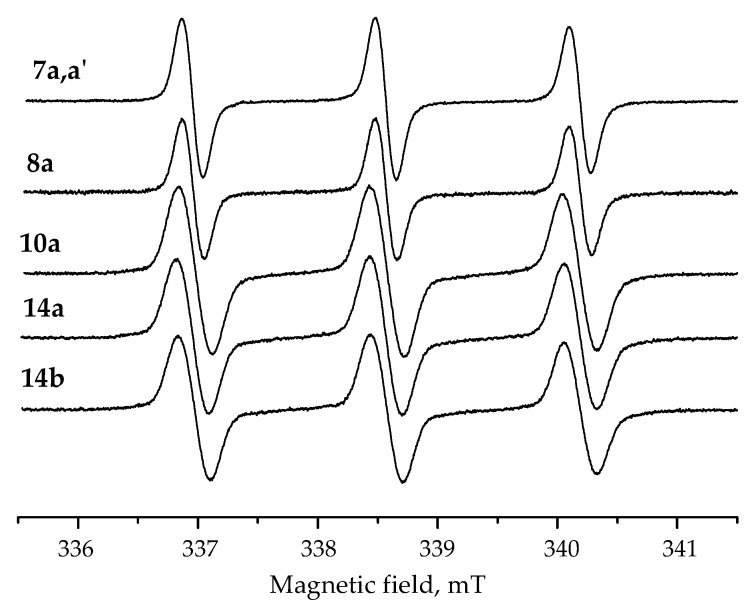
EPR spectra of mixtures **7a,a′** and **8a**, and individual nitroxides **10a**, **14a,** and **14b** (0.2 mM in deoxygenated water). For spectrometer settings, see Section 3.

The EPR spectra of the nitroxides **10a** and **14b** and diastereomeric mixtures **7a,a′**, **8a,** and **14a** are represented in Figure 1 by broadened triplets with no additional resolved hyperfine structure. The broadening obviously resulted from multiple smaller couplings with neighboring γ and δ-hydrogens, indicating some averaging due to relatively free rotation of the alkyl groups. Parameters of EPR spectra, partition coefficients octanol/water and rate constants of reduction with ascorbate of mixtures **7a,a′** and **8a**, and individual nitroxides **10a**, **14a,** and **14b** are listed in the Table 1. In mixtures of **7a,a’** and **8a,** the contribution of the minor component did not exceed 15% and produced no effect on the apparent linewidths. Moreover, the spectrum of **14a**, which is a mixture of two diastereomers in equal ratio, does not show broader lines than spectra of **10a** and **14b**. Linewidths in the spectra of **10a** and **14a,b** were ca. 40% higher than those reported for nitroxides prepared by Lampp et al. [12]; however, they were ca. 40% lower than overall widths of the component of the nitroxide triplet (H_pp_ + *a*_H_) in similar 2-*tert*-butyl-3,4-disubstituted nitroxide [14].

The spectra of nitroxides with ethynyl moiety showed remarkably smaller linewidths than those of similar nitroxides with ethyl groups. All the new nitroxides are lipophilic, but their partition coefficients were significantly lower than those of earlier described β-*tert*-butyl-substituted nitroxides of pyrrolidine series [14]. The nitroxide with two 3-hydroxypropyl groups **14b** showed the lowest partition coefficient in octanol-water mixtures. The **7a,a′** and **10a** are weak acids and their distribution octanol–water mixtures may be accompanied by partial ionization of carboxylic groups. Adjustment of pH of the water phase to 9 leads to redistribution of the nitroxides into water solution with K_p_ < 0.1. Similar to the earlier described pattern [14], *tert*-butyl-substituted nitroxides **10a**, **14a** and **14b** demonstrate very high resistance to reduction. The distant functional groups in the side chains (carboxy or hydroxyl) produce minor effect on the reduction rate. As expected, nitroxides **7a,a′** and **8a** were much stronger oxidants due to the electron-withdrawing effect of ethynyl group. 

## 3. Materials and Methods

### 3.1. General Information

The IR spectra were recorded on a Bruker Vector 22 FT-IR spectrometer (Bruker, Billerica, MA, USA) in KBr pellets (1:150 ratio) or in neat samples (see Figures S1–S13). The 1H NMR spectra were recorded on a Bruker AV 300 (300.132 MHz), AV 400(400.134 MHz) and DRX 500 (500.130 MHz) spectrometers (Bruker, Billerica, MA, USA). 13C NMR spectra were recorded on a Bruker AV 300 (75.467 MHz), AV 400 (100.614 MHz) and DRX 500 (125.758 MHz) spectrometers (see Figures S14–S35). All NMR spectra were acquired for 5–10% solutions in CDCl_3_, (CD_3_)_2_SO or CDCl_3_–CD_3_OD mixtures at 300 K using the signal of the solvent as a standard. HRMS analyses were performed with high-resolution mass spectrometer DFS (Thermo Electron, Waltham, MA, USA). NMR spectra of nitroxides for analysis and structure assignment were recorded after reduction with Zn in CD_3_OD–CF_3_COOH at 65 °C, as described in [13]. HPLC analysis was performed with an Agilent 1100 liquid chromatography system (Agilent Technologies, Santa Clara, CA, USA) equipped with a quaternary pump, online degasser, autosampler, and diode array detector. Chromatographic separations were carried out on a ZORBAX SB-C18 column (150 mm × 4.6 mm, 5.0 μm) by use of binary solvent system MeCN:H_2_O (4:1 *v*/*v*). Flow rate was 0.7 mL/min. Detection was performed at 238 nm.

Reactions were monitored by thin-layer chromatography (TLC) on Merck TLC Silica gel 60 F254 plates. Kieselgel60 (Macherey-Nagel GmbH & Co. KG, Düren, Germany) was utilized as sorbent for the column chromatography.

EPR experiments were performed on an X-band (9.8 GHz) EPR spectrometer ER-200D (Bruker, Billerica, MA, USA). All measurements were performed in a 50 μL glass capillary.

#### 3.1.1. Conditions for Spectral Analysis

Radicals concentrations were 0.2 mM in deoxygenated distilled deionized water. Spectra were fitted with Winsim program to calculate hyperfine constants. g-factor was calculated versus Li:F (g = 2.002293). EPR settings: modulation amplitude, 0.8 G; MW power, 5 mW; time constant, 50 ms; total acquisition time, 3 min.

#### 3.1.2. Conditions for Kinetics Experiments

Radical concentrations were 0.1–0.2 mM in phosphate buffer (5 mM, pH −7.5). Glutathione, 5 mM; ascorbic acid: 100, 200, 300 mM (for nitroxides **10a**, **14a**, **14b**); 33, 66, 100 mM (for **7a**,**b** and **8a**). Kinetics of the decrease in EPR signal of radicals with time was recorded. Total kinetics time was 3 h (nitroxides **10a**, **14a**, **14b**) or 10 min (**7a,a′** and **8a**). Pseudo first-order reaction rate constants were calculated for each ascorbic acid concentration from initial slope (nitroxides **10a**, **14a**, **14b**) or exponential decay rate (**7a,a′** and **8a**) of kinetics. Second-order rate constants were calculated from linear fitting of the pseudo first order rate constants versus ascorbic acid concentration. EPR settings: modulation amplitude, 2.5 G; microwave power, 5 mW; time constant, 20 ms.

#### 3.1.3. Partition Coefficient Measurements 

A solution of a nitroxide (0.1–0.2 mM) in water (1 mL) was prepared. Aliquots of the solution were taken into 50 μL capillary for measurements of amplitude of low field component of EPR spectrum. Thereafter, this aliquot was returned back, required volume octanol (1–5% of water volume) was added, and the mixture was shacked and then shortly centrifuged to separate aqueous and octanol phase. Then, the aliquot of aqueous phase was carefully taken to measure the EPR signal again. This procedure was repeated at least three times for each sample. The amplitude of the intensity of the low field band of the nitroxide EPR spectrum in octanol fraction and the intensity of the initially recorded spectrum in water were used to calculate the partition coefficient. To measure partition coefficient at pH 9 larger volume of octanol was used (100–300% to that of water). EPR settings: modulation amplitude—2.0–2.5 G; microwave power—5 mW; time constant—50 ms.

Compounds **1a,b** and **2** were prepared using literature procedures [19,20,21].

### 3.2. Synthesis

#### 3.2.1. Synthesis of *tert*-Butyl Substituted Nitrones (General Method)

A methyl-4-nitroheptanoate (**1a**) or dimethyl 4-nitroheptanedioate (**1b**) (100 mmol) was added dropwise to freshly prepared solution of sodium methylate in methanol (0.35 M, 40 mL) within 15 min. The yellow solution formed was stirred for 20 min at room temperature, then a solution of 4,4-dimethylpent-1-en-3-one **2** (100 mmol) in 40 mL of dry methanol was added dropwise within 30 min. The reaction mixture was allowed to cool down to room temperature, then heated to reflux for 3 h. (TLC control on SiO_2_, chloroform, stained with phosphomolybdic acid). The pH was adjusted to neutral by addition of glacial acetic acid (ca. 1.5 mL). and the solvent was distilled off in vacuum. The residue was dissolved in ethyl acetate (150 mL) and successively washed with water (70 mL), saturated solution of sodium bicarbonate (100 mL) and again with water (100 mL). The organic layer was dried with anhydrous Na_2_SO_4_ and the solvent was evaporated in vacuum. The residue was dissolved in 30 mL of THF and mixed with aqueous solution ammonium chloride (1.7 M, 60 mL). The resulting solution was cooled to 7 °C, and zinc dust (24.0 g, 370 mmol) was added by small portion maintaining the temperature in the range of 7–15 °C. The reaction mixture was stirred for 1 h at 5 °C and then 1 h at room temperature. Inorganic precipitate was filtered off and washed with ethanol (200 mL). The filtrate was concentrated in vacuum and the residue was dissolved in water (50 mL). Water solution was extracted with diethyl ether (50 mL) and organic layer was discarded. Water layer was saturated by NaCl and extracted with CHCl_3_ (3 × 100 mL). The combined organic extracts were dried with anhydrous Na_2_SO_4_. The solvent was evaporated in vacuum, the crude residue was purified by column chromatography (SiO_2_, chloroform-methanol 40:1 mixture as an eluent, detected under UV lamp) to give **4a** or **4b**.

Methyl 3-(5-*tert*-butyl-2-ethyl-1-oxido-3,4-dihydro-*2H*-pyrrol-2-yl)propanoate (**4a**): yield 15.3 g (60%). Colorless oil. HRMS (EI/DFS) *m*/*z* [M]^+^ calcd. for (C_14_H_25_NO_3_)^+^: 255.1829; found: 255.1831. IR (neat) ν_max_: 1737 (C=O), 1573 (C=N). ^1^H NMR (500 MHz; CDCl_3_, δ): 0.77 (t, *J*_t_ = 7.4 Hz, 3H), 1.22 (s, 9H, *t*-Bu), 1.41–1.50 (m, 1H), 1.73–1.82 (m, 2H), 1.83–1.92 (m, 2H), 2.07–2.15 (m, 1H), 2.16–2.28 (m, 2H), 2.51 (dd, *J*_d1_ = 7.6 Hz, *J*_d2_ = 7.2 Hz, 2H), 3.58 (s, 3H, OCH_3_). ^13^CNMR (125 MHz; CDCl_3_, δ): 7.29, 23.98, 25.26, 27.31, 28.23, 30.50, 32.67, 33.33, 51.38, 79.25, 150.67, 173.3.

Dimethyl 3,3′-(5-*tert*-butyl-1-oxido-3,4-dihydro-*2H*-pyrrole-2,2-diyl)dipropanoate (**4b**): yield 17.8 g (57%). Colorless oil. HRMS (EI/DFS) *m*/*z* [M]^+^ calcd. for (C_16_H_27_NO_5_)^+^: 313.1884; found: 313.1880. IR (neat) ν_max_: 1737 (C=O), 1573 (C=N). ^1^H NMR (400 MHz; CDCl_3_, δ): 1.15 (s, 9H, *t*-Bu), 1.71–1.82 (m, 4H), 2.02–2.11 (m, 2H), 2.11–2.19 (m, 4H), 2.45–2.51 (m, 2H), 3.52 (s, 6H, OCH_3_). ^13^CNMR (100 MHz; CDCl_3_, δ): 24.44, 25.15, 27.13, 28.07, 32.53, 33.35, 51.49, 78.04, 151.06, 173.02.

#### 3.2.2. 3-(5-*tert*-Butyl-2-ethyl-1-oxido-3,4-dihydro-2*H*-pyrrol-2-yl)propanoic Acid (**5a**)

An aqueous solution of sodium hydroxide (3.2 M, 50 mL) was added to the solution of **4a** (10.2 g, 40 mmol) in methanol (50 mL), and the resulting homogeneous mixture was left at room temperature for 24 h. Methanol was distilled off in vacuum, the water solution was shaken with diethyl ether (50 mL), and the organic layer was discarded. Chloroform (70 mL) was added to the water layer and 2 M aqueous solution of H_2_SO_4_(40 mL) was added to the mixture upon stirring. The organic layer was separated, and the water layer was extracted with CHCl_3_ (2 × 50 mL). Combined organic extracts were dried with anhydrous Na_2_SO_4_, the solvent was evaporated in vacuum, the crude residue was treated with diethyl ether (30 mL), and and white crystalline precipitate of **5a** was filtered off, yield 8.5 g, (91%), m.p. 160.6–162.0 °C (ethyl acetate). Found: C, 64.91; H, 9.31; N, 5.93; calcd. For C_13_H_23_NO_3_: C, 64.70; H, 9.61; N, 5.80%; IR (KBr) ν_max_: 1726 (C=O), 1594 (C=N); ^1^H NMR (500 MHz; CDCl_3_, δ): 0.79 (t, *J*_t_ = 7.4 Hz, 3H), 1.26 (s, 9H, *t*-Bu), 1.46–1.55 (m, 1H), 1.76–1.86 (m, 2H), 1.87–1.96 (m, 2H), 2.09–2.21 (m, 2H), 2.26–2.34 (m, 1H), 2.54–2.66 (m, 2H), 11.16 (br. S, 1H). ^13^CNMR (125 MHz; CDCl_3_, δ): 7.30, 24.01, 25.55, 28.03, 28.76, 30.55, 32.10, 33.84, 80.39, 157.62, 174.74.

#### 3.2.3. 3,3′-(5-*tert*-Butyl-1-oxido-3,4-dihydro-2*H*-pyrrole-2,2-diyl)dipropanoic Acid (**5b**)

An aqueous solution of sodium hydroxide (3.2 M, 5 mL) was added to the solution of **4b** (1.25 g, 4 mmol) in methanol (5 mL). The resulting homogeneous solution was allowed to stand at room temperature for 24 h, methanol was distilled off in vacuum pressure and 2M solution of H_2_SO_4_ (4.5 mL) was added to the residue upon stirring. The formed precipitate of **5b** was filtered off and successively washed with ice-cold water (2 mL) and diethyl ether (10 mL) and dried in vacuum, yield 1.0 g (95%). White crystalline solid, m.p. 223.3–224.5 °C (ethanol). Found: C, 59.22; H, 8.29; N, 4.78; calcd. For C_14_H_23_NO_5_: C, 58.93; H, 8.12; N, 4.91%; IR (KBr) ν_max_: 1720 (C=O), 1594 (C=N). ^1^H NMR (300 MHz; (CD_3_)_2_SO, δ): 1.20 (s, 9H, *t*-Bu), 1.62–1.76 (m, 2H), 1.80–2.05 (m, 6H), 2.16–2.32 (m, 2H), 2.54–2.67 (m, 2H). ^13^CNMR (75 MHz; (CD_3_)_2_SO, δ): 23.66, 24.89, 26.15, 26.78, 28.00, 32.71, 33.07, 77.91, 150.65, 174.17

#### 3.2.4. 2-*tert*-Butyl-5-(2-carboxyethyl)-5-ethyl-2-ethynylpyrrolidine-1-oxyl (**7a,a′**)

A powder of **5a** (3.6 g, 15 mmol) was added to a 0.5–1 M solution of ethynyl-magnesium bromide in THF (250 mL) upon stirring. The mixture was allowed to stand at room temperature for 168 h (TLC control on SiO_2_, ethyl acetate: methanol: acetic acid 100:10:1, detected under UV lamp), then quenched with water (20 mL) and acidified with saturated aqueous sodium bisulfate solution (150 mL) to pH 3–4. The organic layer was separated, and the water phase was extracted with ethyl acetate (2 × 100 mL). The combined organic layers were dried with anhydrous Na_2_SO_4_. The solvent was evaporated in vacuum and the crude residue was dissolved in methanol (70 mL) and basified with sodium hydroxide solution (1 M, 20 mL). Methylene blue (6 mg, 0.02 mmol) was added to the mixture and the air was bubbled until the solution turned dark blue. The methanol was distilled off in vacuum, and the remaining aqueous solution was washed with diethyl ether (30 mL), acidified with saturated aqueous sodium bisulfate solution (20 mL) to pH < 4, and extracted with ethyl acetate (3 × 50 mL). The organic phase was dried with Na_2_SO_4_ and the solvent was evaporated in vacuum. The residue was purified by column chromatography (SiO_2_, eluent ethyl acetate: methanol: acetic acid 100:10:1) to give of **7a,a′**, yield 2.40 g (60%), yellow crystalline solid, m.p. 109.9–112.3 °C (hexane: ethyl acetate 5:1). Found: C, 67.66; H, 9.04; N, 5.22; calcd. for C_15_H_24_NO_3_: C, 67.64; H, 9.08; N, 5.26%; IR (KBr) ν_max_: 3309, 2983, 2969, 2956, 1718, 1214, 619. ^1^H NMR(400 MHz; CD_3_OD/CDCl_3_, Zn/CF_3_COOH system δ): 0.63 (t, *J*_t_ = 7.5 Hz, 3H), 0.86 (s, 9H, *t*-Bu), 1.34–1.53 (m, 1H), 1.53–1.67 (m, 2H), 1.71–1.85 (m, 3H), 1.89–2.06 (m, 1H), 2.06–2.16 (m, 1H), 2.23–2.29 (m, 2H), 2.75 (s, 1H). Signals of minor isomer: 0.50 (t, *J*_t_ = 7.5 Hz, 3H), 0.67 (s, 9H, *t*-Bu), 2.64 (s, 1H).

#### 3.2.5. 2-*tert*-Butyl-5-ethyl-2-ethynyl-5-(3-methoxy-3-oxopropyl)pyrrolidine-1-oxyl (**8a**)

A solution of **7a** (1.0 g, 3.76 mmol) in diethyl ether (10 mL) was slowly added to the 0.4 M solution (40 mL) of diazomethane in diethyl ether at 0 °C. The reaction mixture was stirred for 1 h at this temperature. The reaction was monitored by TLC (SiO_2_, ethyl acetate: methanol: acetic acid 200:20:1 mixture; detected under UV lamp). Then, acetic acid (2 mL) was slowly added to remove excess of diazomethane, and the resulting solution was washed with 10% aqueous sodium bicarbonate (2 × 25 mL) and water (1 × 25 mL). The organic phase was dried with Na_2_SO_4,_ and the solvent was evaporated in vacuum. The residue was purified by column chromatography (SiO_2_, ethyl acetate as an eluent, detected under UV lamp) to give **8a**, yield 1.0 g (95%), yellow crystalline solid, m.p. 65.4–66.2 °C (hexane). Found: C, 68.79; H, 9.22; N, 4.97; calcd. for C_16_H_26_NO_3_: C, 68.54; H, 9.35; N, 5.00%; IR (KBr) ν_max_: 3259 (≡C-H), 1727 (C=O). ^1^H NMR (500 MHz; CD_3_OD/CDCl_3_, Zn/CF_3_COOH system δ): 0.97 (t, *J*_t_ = 7.5 Hz, 3H), 1.20 (s, 9H, *t*-Bu), 1.86–2.26 (m, 6H), 2.26–2.47 (m, 2H), 2.60–2.68 (m, 2H), 3.12 (s, 1H), 3.74 (s, 3H). 

#### 3.2.6. 2-*tert*-Butyl-5-(2-carboxyethyl)-2,5-diethylpyrrolidine-1-oxyl (**10a**)

A solution of **8a** (1.0 g, 3.6 mmol) in methanol (10 mL) was placed in the reaction vessel equipped with magnetic stirrer and connection line to gasometer filled with hydrogen. The catalyst (Pd/C, 4%, 30 mg) was added, and the system was purged with hydrogen and closed. The mixture was vigorously stirred until hydrogen absorption ceased (ca. 7 h, 0.22 L of hydrogen absorbed), then the catalyst was filtered off and washed with methanol. Filtrate was mixed with 2M aqueous solution of sodium hydroxide (10 mL) and allowed to stand at room temperature for 10 h. The solution turned yellow. Methanol was distilled off in vacuum, the remaining water solution was shaken with diethyl ether (15 mL) and organic layer was discarded. Chloroform (20 mL) was added to the mixture was acidified with saturated aqueous sodium bisulfate solution (10 mL) to pH < 4 upon stirring. Organic layer was separated and water layer was extracted with chloroform (20 mL). Combined extract was dried with Na_2_SO_4_ and the solvent was evaporated in vacuum. The residue was purified by column chromatography (SiO_2_, ethyl acetate: methanol: aceticacid100:10:1 mixture as an eluent) to give 10a, yield 0.90 g (92%), yellow crystalline solid,. m.p. 101–102 °C dec. (hexane). Found: C, 66.81; H, 10.49; N, 5.14; calcd. for C_15_H_28_NO_3_: C, 66.63; H, 10.44; N, 5.18%; IR (KBr) ν _max_: 1704 (C=O). ^1^H NMR (400 MHz; CD_3_OD/CDCl_3_, Zn/CF_3_COOH system δ): 0.71 (t, *J*_t_ = 7.4 Hz, 3H), 0.84 (t, *J*_t_ = 7.5 Hz, 3H), 0.88 (s, 9H, *t*-Bu), 1.43–1.60 (m, 3H), 1.63–1.71 (m, 1H), 1.74–2.03 (m, 6H), 2.29–2.45 (m, 2H).

#### 3.2.7. Synthesis of *tert*-Butyl Substituted Nitrones, Containing 3-Hydroxypropyl Moiety (General Method)

A solution of corresponding nitrone **4a,b** (10 mmol) in anhydrous diethyl ether (10 mL) was added dropwise within 20 min to a suspension of lithium aluminum hydride (1.14 g, 30 mmol) in anhydrous diethyl ether (30 mL) upon stirring. After the spontaneous boiling of the reaction mixture ceased, it was heated to reflux and stirred for 4 h. The reaction was monitored by TLC (SiO_2_, chloroform-methanol 10:1 mixture; stained with Dragendorff’s reagent). The reaction mixture was cooled in an ice bath, and excess of lithium aluminum hydride was quenched with water (5 mL). The ether layer was separated by decantation and residue was washed with ether (2 × 50 mL). The combined organic layers were dried with anhydrous Na_2_SO_4_, the solvent was evaporated in vacuum and the crude residue was stirred with chloroform (50 mL) and manganese dioxide (3.44 g, 40 mmol) at room temperature for 3 h. The reaction was monitored by TLC (SiO_2_, chloroform-methanol 10:1 mixture; stained with Dragendorff’s reagent). Manganese oxides were filtered off, precipitate was washed with chloroform (30 mL) and methanol (30 mL), the filtrate was evaporated in vacuum, and the residue was purified by column chromatography (SiO_2_, chloroform-methanol 15:1 mixture as an eluent, detected under UV lamp).

3-(5-*tert*-Butyl-2-ethyl-1-oxido-3,4-dihydro-*2H*-pyrrol-2-yl)-1-propanol (**11a**): 0.95 g, yield 42%. Colorless oil. HRMS (EI/DFS) *m*/*z* [M]^+^ calcd. for (C_13_H_25_NO_2_)^+^: 227.1880; found:227.1882. IR (neat) ν_max_: 3346 (O–H), 1583 (C = N). ^1^H NMR (400 MHz; CDCl_3_, δ): 0.70 (t, *J*_t_ = 7.4 Hz, 3H), 1.17 (s, 9H, *t*-Bu), 1.22–1.34 (m, 1H), 1.34–1.51 (m, 3H), 1.73–1.89 (m, 4H), 2.45–2.51 (m, 2H), 3.46 (dt, *J*_t_ = 6.2 Hz, *J*_d_ = 1.8 Hz, 2H), 4.21 (br.s, 1H); ^13^C NMR (100 MHz; CDCl_3_, δ): 7.27, 23.63, 25.24, 26.05, 27.56, 30.98, 33.32, 33.53, 61.62, 80.12, 152.49.

3,3′-(5-*tert*-Butyl-1-oxido-3,4-dihydro-*2H*-pyrrole-2,2-diyl)di(1-propanol) (**11b**): 1.64 g, yield 64%. White crystalline solid, m.p. 136.7–137.2 °C (ethyl acetate). Elemental analysis: found: C, 65.33; H, 10.57; N, 5.44; calcd. for C_14_H_27_NO_3_: C, 65.53; H, 10.78; N, 5.51%; IR (KBr) ν_max_: 3344 (O–H), 1600 (C=N). ^1^H NMR (500 MHz; CDCl_3_, δ): 1.20 (s, 9H, *t*-Bu), 1.23–1.33 (m, 2H), 1.42–1.55 (m, 4H), 1.81–1.91 (m, 4H), 2.52–2.56 (m, 2H), 3.49 (t, *J*_t_ = 6.2 Hz, 4H), 4.38 (br.s, 2H); ^13^C NMR (125 MHz; CDCl_3_, δ): 24.19, 25.35, 26.02, 27.75, 33.50, 34.20, 61.60, 79.87, 154.38.

#### 3.2.8. Reaction of Nitrones **11a** and **11b** with 2,2-Dimethoxypropane (General Method)

A mixture of **11a** or **11b** (10 mmol), 2,2-dimethoxypropane (48.9 mL, 400 mmol), PPTS (502 mg, 2 mmol), molecular sieves 4 Å (10 g) and anhydrous chloroform (50 mL) was stirred at room temperature for 48 h. The reaction was monitored by TLC (SiO_2_, chloroform-methanol 15:1 mixture; detected under UV lamp). After that, sieves were filtered off, and the filtrate was washed with aqueous sodium bicarbonate saturated solution (3 × 30 mL) and dried with anhydrous Na_2_SO_4_. The solvent was evaporated in vacuum, and the crude residue was purified by column chromatography (SiO_2_, chloroform-methanol 20:1 mixture as an eluent, detected under UV lamp) to give desired nitrone.

5-*tert*-Butyl-2-ethyl-2-[3-(1-methoxy-1-methylethoxy)propyl]-3,4-dihydro-*2H*-pyrrole 1-oxide (**12a**): 2.48 g, yield 83%. Colorless oil. HRMS (EI/DFS) *m*/*z* [M]^+^ calcd. for (C_17_H_33_NO_3_)^+^:299.2455; found: 299.2465. IR (neat) ν_max_: 1578 (C = N). ^1^H NMR (500 MHz; CDCl_3_, δ): 0.75 (t, *J*_t_ = 7.4 Hz, 3H), 1.21 (s, 9H, *t*-Bu), 1.23 (s, 6H), 1.52 (dt, *J*_t_ = 13.2 Hz, *J*_d_ = 4.4 Hz, 1H), 1.77 (dt, *J*_t_ = 13.2 Hz, *J*_d_ = 4.4 Hz, 1H), 1.81–1.88 (m, 3H), 2.48–2.53 (m, 2H), 3.09 (s, 3H), 3.22–3.29 (m, 1H), 3.32–3.37 (m, 1H); ^13^C NMR (125 MHz; CDCl_3_, δ): 7.37, 23.69, 23.90, 24.20, 25.31, 27.48, 30.74, 33.30, 34.63, 48.21, 60.37, 79.89, 99.53, 150.58.

5-*tert*-Butyl-2,2-bis [3-(1-methoxy-1-methylethoxy)propyl]-3,4-dihydro-*2H*-pyrrole 1-oxide (**12b**): 3.2 g, yield 80%. Colorless oil. HRMS (EI/DFS) *m*/*z* [M]^+^ calcd. for (C_22_H_43_NO_5_)^+^:401.3136; found: 401.3138.IR (neat) ν_max_: 1573 (C = N). ^1^H NMR (500 MHz; CDCl_3_, δ): 1.18 (s, 9H, *t*-Bu), 1.20 (s, 12H), 1.27–1.44 (m, 4H), 1.51 (dt, *J*_t_ = 12.8 Hz, *J*_d_ = 4.3 Hz, 2H), 1.75 (dt, *J*_t_ = 13.0 Hz, *J*_d_ = 4.5 Hz, 2H), 1.83–1.87 (m, 2H), 2.46–2.51 (m, 2H), 3.05 (s, 6H), 3.19–3.25 (m, 2H), 3.28–3.34 (m, 2H); ^13^C NMR (125 MHz; CDCl_3_, δ): 23.58, 24.13, 24.44, 25.21, 27.35, 33.21, 34.75, 48.13, 60.24, 79.29, 99.46, 150.31.

#### 3.2.9. Reaction of Nitrones **12a** and **12b** with Ethyllithium (General Method)

A solution of ethyllithium in *n*-pentane (0.7 M, 70 mL) was slowly added dropwise to a solution of **12a** or **12b** (6 mmol) in dry benzene (10 mL) upon stirring under argon. The mixture was stirred for 20 h at room temperature, cooled in the ice bath, and quenched with water (20 mL). The organic layer was separated and water layer was extracted with diethyl ether (2 × 30 mL). Combined extracts were dried with Na_2_SO_4_ and the solvent was evaporated in vacuum. The crude residue was dissolved in methanol (70 mL) and basified with aqueous solution of sodium hydroxide (1 M, 20 mL). Methylene blue (6 mg, 0.02 mmol) was added to the mixture, and the air was bubbled until the solution had turned dark blue. The methanol was distilled off in vacuum, and the residue was extracted with diethyl ether (3 × 30 mL). Combined extract was dried with Na_2_SO_4_ and the solvent was evaporated in vacuum. The residue was dissolved in the 30 mL of mixture methanol:water (1:1), and PPTS (500 mg) was added. The mixture was stirred for 1 h at room temperature, methanol was distilled off in vacuum and residue was extracted with diethyl ether (3 × 30 mL). Combined extract was dried with Na_2_SO_4_ and the solvent was evaporated in vacuum. The residue was purified by column chromatography (SiO_2_, chloroform-methanol 30:1 or hexane-ethyl acetate1:1 mixture as an eluent, detected under UV lamp) to give desired nitroxide.

2-*tert*-Butyl-2,5-diethyl-5-(3-hydroxypropyl)pyrrolidine-1-oxyl (**14a**):yield 1.04 g (68%), yellow oil. HRMS (EI/DFS) *m*/*z* [M]^+^ calcd. for (C_15_H_30_NO_2_)^+^: 256.2271; found: 256.2273. IR (neat) ν_max_: 3425 (O–H). ^1^H NMR (400 MHz; CD_3_OD/CDCl_3_, Zn/CF_3_COOH system δ): 0.74 (q, *J*_q_ = 7.3 Hz, 3H), 0.85 (t, *J*_t_ = 7.5 Hz, 3H, signals of the first isomer), 0.87 (s, 9H, *t*-Bu), 0.88 (t, *J*_t_ = 7.5 Hz, 3H, signals of the second isomer), 1.37–1.99 (m, 12H), 3.24–3.37 (m, 1H), 3.44–3.55 (m, 1H).

2-*tert*-Butyl-2-ethyl-5,5-bis(3-hydroxypropyl)pyrrolidine-1-oxyl (**14b**):yield 0.91 g (53%), yellow crystalline solid,. m.p. 93.0–95.2 °C (hexane-ethyl acetate 1:1). Found: C, 67.44; H, 11.19; N, 5.06; calcd. for C_16_H_32_NO_3_: C, 67.09; H, 11.26; N, 4.89%; IR (KBr) ν_max_: 3332 (O–H). ^1^H NMR (400 MHz; CD_3_OD/CDCl_3_, Zn/CF_3_COOH system δ): 0.77 (t, *J*_t_ = 7.5 Hz, 3H), 0.78 (s, 9H, *t*-Bu), 1.20–1.37 (m, 4H), 1.40–1.62 (m, 4H), 1.62–1.74 (m, 4H), 1.74–1.93 (m, 2H), 3.22–3.42 (m, 4H).

## 4. Conclusions

We showed that 3,4-unsubstituted 2-*tert*-butyl-2-ethylpyrrolidine-1-oxyls can be prepared from the corresponding 2-*tert*-butyl-1-pyrroline-1-oxides via either the direct addition of ethyllithium or treatment with ethynylmagnesium bromide with subsequent successive hydrogenation of terminal ethynyl group. Nitroxides with 2-*tert*-butyl group adjacent to the N-O· moiety demonstrated very high stability to reduction with ascorbate. Distant polar groups, such as carboxylic or hydroxy, do not affect the rate of reduction of the nitroxide much, but increase solubility. New 2-*tert*-butyl-substituted nitroxides **10a**, **14a** and **14b** are currently the most stable against the reduction of water-soluble nitroxides.

Removal of the substituents from the 3,4-positions of the ring in sterically shielded pyrrolidine nitroxides allows for avoiding large splitting on γ-hydrogens in the EPR spectra, but the line widths are still high. 

## Figures and Tables

**Table 1:** Parameters of EPR spectra in water, partition coefficients octanol/water, and reduction rate constants with ascorbate.

Nitroxide	g-Factor	Hp-p, mT (Center)	*a*_N_, mT	Partition Coefficient	k_red_, M^−1^ s^−1^
**7a,a′**	2.00571 ± 2	0.17 ± 0.005	1.61 ± 0.005	150	0.2 ± 0.05
**8a**	2.00571 ± 2	0.17 ± 0.005	1.61 ± 0.005	600	0.6 ± 0.1
**10a**	2.00563 ± 2	0.28 ± 0.005	1.61 ± 0.005	240	(4 ± 1) × 10^−5^
**14a**	2.00560 ± 2	0.26 ± 0.005	1.61 ± 0.005	400	(3 ± 1) × 10^−5^
**14b**	2.00562 ± 2	0.26 ± 0.005	1.61 ± 0.005	70	(5 ± 1) × 10^−5^

**Figure A1:**
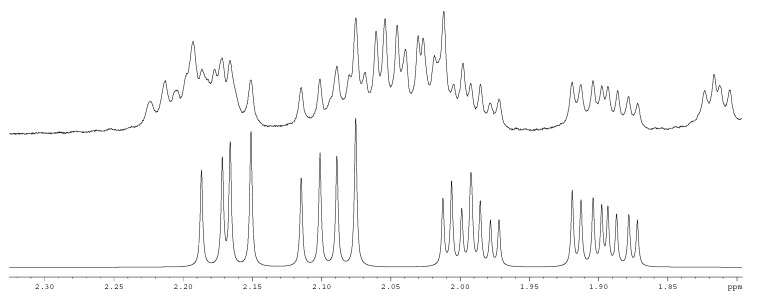
(top) Real and (bottom) simulated (fragment of pyrrolidine ring) ^1^H-NMR spectra.

**Figure A2:**
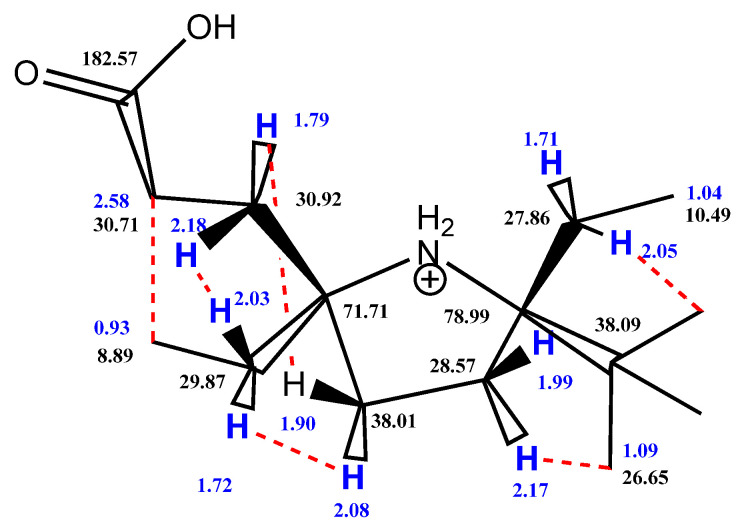
Assignment of signals of ^13^C-NMR (black) and ^1^H NMR (blue) for **10a_*red***. Slashed red lines show through-space interactions in ^1^H,^1^H–NOESY spectrum.

## Data Availability

Data are contained within the article or Supplementary Material.

## Supplementary Materials

The following supporting information can be downloaded at: https://www.mdpi.com/article/10.3390/molecules27061922/s1, Figures S1–S13: IR spectra of **4a,b**, **5a,b**, **7a,a′**, **8a**, **10a**, **11a,b**, **12a,b**, **14a,b**; Figures S14–S39: NMR spectra of **4a,b**, **5a,b**, **7a,a′_*red***, **8a_*red***, **10a_*red***, **11a,b**, **12a,b**, **14a_*red*, 14b_*red***; Figure S40: HPLC analysis data of **14a**.

## Sample Availability

Samples of the compounds **10a**, **14a** and **14b** are available from the authors.

## Appendix A

The 1D and 2D NMR spectra of **10a_*red*** were recorded from a mixed CD_3_OD/CDCl_3_ solution at 303K on a Bruker Avance III 500 FT-spectrometer with working frequencies 500.13 and 125.76 MHz for ^1^H and ^13^C, respectively. ^1^H and ^13^C NMR chemical shifts are reported in ppm of the δ scale and referred to signals of the CD_3_OD (3.31 ppm for residual protons for ^1^H and 49.15 for ^13^C NMR spectra). Signals were assigned using 2D (HSQC, HMBC, COSY) NMR techniques.

The presence of two asymmetric centers causes nonequivalent of hydrogen atoms in the methylene groups of the molecule. Two methylene groups of pyrrolidine were identified using ^1^H, ^13^C-HSQC, and HMBC techniques. Two of four signals overlapped with other multiplets, but the approximate value of J_HH_-coupling constant can be elicited for them: for the first methylene group: δ_C_ = 28.57 ppm, δ_H_ = 1.99 (*J*_1_ = 10.4 Hz, *J*_2_ = 6.7 Hz, *J*_3_ = 3.3 Hz) and δ_H_ = 2.17 (*J*_1_ = 10.4 Hz, *J*_2_ = 7.6 Hz) ppm; for the second: δ_C_ = 38.01 ppm, δ_H_ = 1.90 (*J*_1_ = 13 Hz, *J*_2_ = 7.6 Hz, *J*_3_ = 3.3 Hz) and δ_H_ = 2.08 (*J*_1_ = 13 Hz, *J*_2_ = 6.7 Hz). The third *J*-constants were not observed for signals at δ_H_ = 2.17 and δ_H_ = 2.08 ppm, so their values were approximately equal to 0 Hz. So, synclinal orientation for protons pairs with angle close to 90°at 1.90 and 1.99 ppm on the one hand, and 2.08 and 2.17 ppm on the other hand can be suggested.

Weak cross-peaks in the ^1^H,^1^H–NOESY spectrum showed interaction of the methyl group at 1.09 ppm with protons at 2.17 ppm (methylene group of pyrrolidine ring) and at 2.05 ppm (ethyl group). Some cross-peaks for the other side of molecule were also detected: between signals at 0.93 and 2.58 ppm, 1.72 and 2.08 ppm, 1.79 and 1.90 ppm, 2.03 and 2.18 ppm. So, we can conclude that *tert*-butyl and ethyl groups were oriented in the same plane with hydrogen atoms at 2.08 and 2.17 ppm of the pyrrolidine cycle.
