# Supplementary material for: 3,4-Unsubstituted 2-tert-Butyl-pyrrolidine-1-oxyls with Hydrophilic Functional Groups in the Side Chains
Source: Molecules. 2022 Mar 16;27(6):1922. doi: 10.3390/molecules27061922 (PMC8948954; doi:10.3390/molecules27061922)
Supplement: Supplementary file 1 [file molecules-27-01922-s001.zip › molecules-1623685-supplementary.pdf]

3,4-Unsubstituted 2-*tert*-butyl-pyrrolidine-1-oxyls with hydrophilic functional groups in the side chains

Andrey I. Taratayko [1,*], Yurii I. Glazachev [2], Ilia V. Eltsov [3], Elena I. Chernyak[1] and Igor A. Kirilyuk[1]

[1] N.N. Vorozhtsov Institute of Organic Chemistry SB RAS, Academician Lavrentiev Ave. 9, Novosibirsk 630090, Russia; taratayk@nioch.nsc.ru
[2] Voevodsky Institute of Chemical Kinetics and Combustion SB RAS, Institutskaya 3, Novosibirsk 630090, Russia
[3] Department of Natural Sciences, Novosibirsk State University, Pirogova Str. 1, Novosibirsk 630090, Russia
* Correspondence: taratayk@nioch.nsc.ru

Supporting Information
Table of Contents

1. IR spectral data 3
 1.1 Methyl 3-(5-*tert*-butyl-2-ethyl-1-oxido-3,4-dihydro-*2H*-pyrrol-2-yl)propanoate (**4a**) 3
 1.2 Dimethyl 3,3'-(5-*tert*-butyl-1-oxido-3,4-dihydro-*2H*-pyrrole-2,2-diyl)dipropanoate (**4b**)....3
 1.3 3-(5-*tert*-Butyl-2-ethyl-1-oxido-3,4-dihydro-*2H*-pyrrol-2-yl)propanoic acid (**5a**) 4
 1.4 3,3'-(5-*tert*-Butyl-1-oxido-3,4-dihydro-*2H*-pyrrole-2,2-diyl)dipropanoic acid (**5b**) 4
 1.5 2-*tert*-Butyl-5-(2-carboxyethyl)-5-ethyl-2-ethynylpyrrolidine-1-oxyl (**7a,a'**) 5
 1.6 2-*tert*-Butyl-5-ethyl-2-ethynyl-5-(3-methoxy-3-oxopropyl)pyrrolidine-1-oxyl (**8a**) 5
 1.7 2-*tert*-Butyl-5-(2-carboxyethyl)-2,5-diethylpyrrolidine-1-oxyl (**10a**) 6
 1.8 3-(5-*tert*-Butyl-2-ethyl-1-oxido-3,4-dihydro-*2H*-pyrrol-2-yl)-1-propanol (**11a**) 6
 1.9 3,3'-(5-*tert*-Butyl-1-oxido-3,4-dihydro-*2H*-pyrrole-2,2-diyl)di(1-propanol) (**11b**)......7
 1.10 5-*tert*-Butyl-2-ethyl-2-[3-(1-methoxy-1-methylethoxy)propyl]-3,4-dihydro-*2H*-pyrrole 1-oxide (**12a**) 7
 1.11 5-*tert*-Butyl-2,2-bis[3-(1-methoxy-1-methylethoxy)propyl]-3,4-dihydro-*2H*-pyrrole 1-oxide (**12b**) 8
 1.12 2-*tert*-Butyl-2-ethyl-5,5-bis(3-hydroxypropyl)pyrrolidine-1-oxyl (**14a**) 8
 1.13 2-*tert*-Butyl-2-ethyl-5,5-bis(3-hydroxypropyl)pyrrolidine-1-oxyl (**14b**) 9
2. ^{1}H and ^{13}C NMR spectral data 10
 2.1 Methyl 3-(5-*tert*-butyl-2-ethyl-1-oxido-3,4-dihydro-*2H*-pyrrol-2-yl)propanoate (**4a**) 10
 2.2 Dimethyl 3,3'-(5-*tert*-butyl-1-oxido-3,4-dihydro-*2H*-pyrrole-2,2-diyl)dipropanoate (**4b**)..11
 2.3 3-(5-*tert*-Butyl-2-ethyl-1-oxido-3,4-dihydro-*2H*-pyrrol-2-yl)propanoic acid (**5a**) 12
 2.4 3,3'-(5-*tert*-Butyl-1-oxido-3,4-dihydro-*2H*-pyrrole-2,2-diyl)dipropanoic acid (**5b**) 13
 2.5 2-*tert*-Butyl-5-(2-carboxyethyl)-5-ethyl-2-ethynylpyrrolidinium trifluoroacetate (mixture of diastereomers) (**7a,a'_*red***) 14
 2.6 2-*tert*-Butyl-5-ethyl-2-ethynyl-5-(3-methoxy-3-oxopropyl)pyrrolidinium trifluoroacetate (**8a_red**) 14
 2.7 2-*tert*-Butyl-5-(2-carboxyethyl)-2,5-diethylpyrrolidinium trifluoroacetate (**10a_*red***) 15

2.8 3-(5-*tert*-Butyl-2-ethyl-1-oxido-3,4-dihydro-*2H*-pyrrol-2-yl)-1-propanol (**11a**) 18

2.9 3,3'-(5-*tert*-Butyl-1-oxido-3,4-dihydro-*2H*-pyrrole-2,2-diyl)di(1-propanol) (**11b**).............19

2.10 5-*tert*-Butyl-2-ethyl-2-[3-(1-methoxy-1-methylethoxy)propyl]-3,4-dihydro-*2H*-pyrrole 1-oxide (**12a**) 20

2.11 5-*tert*-Butyl-2,2-bis[3-(1-methoxy-1-methylethoxy)propyl]-3,4-dihydro-*2H*-pyrrole 1-oxide (**12b**)...21

2.12 2-*tert*-Butyl-2,5-diethyl-5-(3-hydroxypropyl)pyrrolidinium trifluoroacetate (mixture of daistereomers) (**14a_*red***) 22

2.13 2-*tert*-Butyl-2-ethyl-5,5-bis(3-hydroxypropyl)pyrrolidinium trifluoroacetate (**14b_*red***).22

3. HPLC analysis 23

3.1 HPLC analysis of 2-*tert*-Butyl-2-ethyl-5,5-bis(3-hydroxypropyl)pyrrolidine-1-oxyl (**14a**) 23

1. IR spectral data

1.1 Methyl 3-(5-*tert*-butyl-2-ethyl-1-oxido-3,4-dihydro-*2H*-pyrrol-2-yl)propanoate (**4a**)

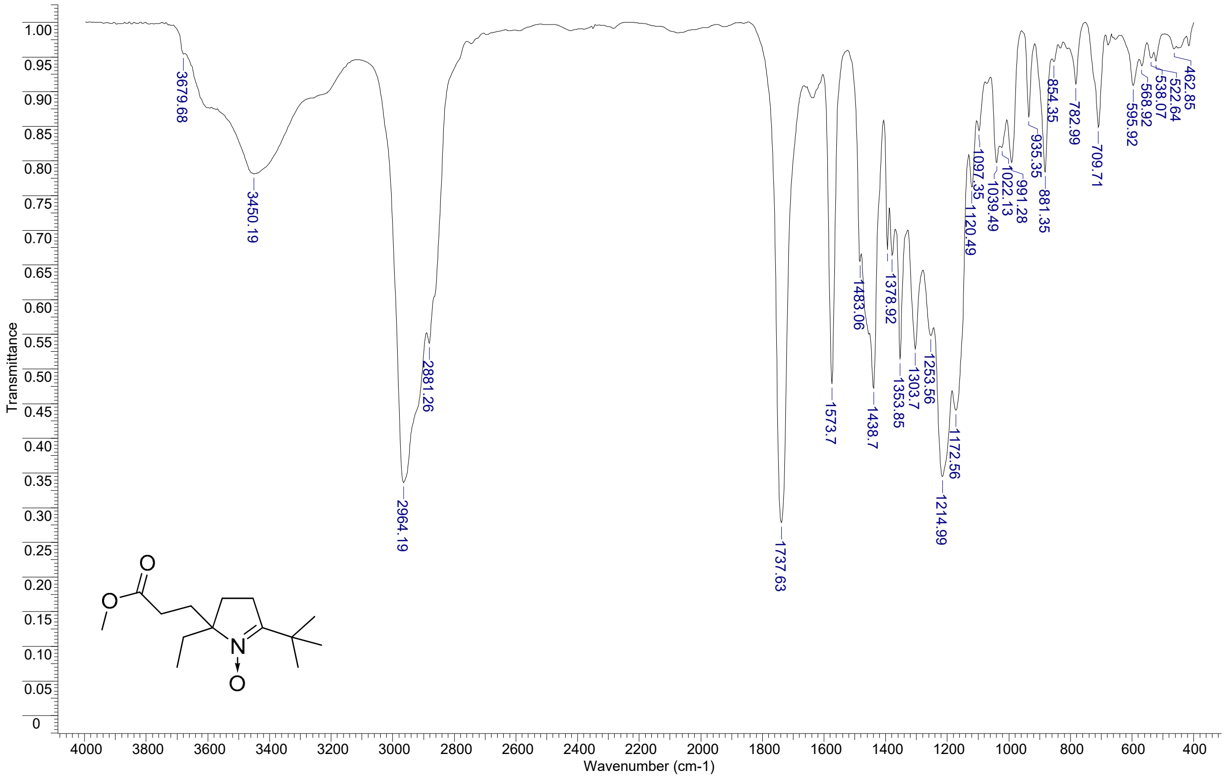

Figure S1. IR spectrum of **4a** (neat)

1.2 Dimethyl 3,3'-(5-*tert*-butyl-1-oxido-3,4-dihydro-*2H*-pyrrole-2,2-diyl)dipropanoate (**4b**)

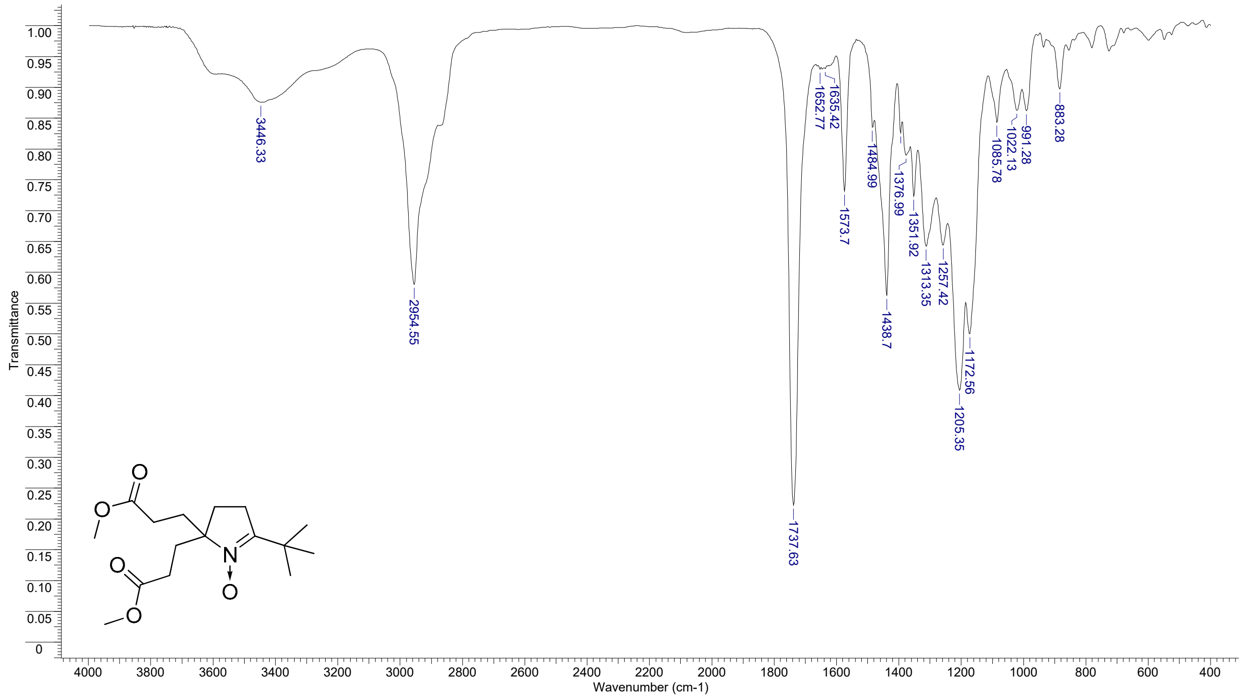

Figure S2. IR spectrum of **4b** (neat)

1.3 3-(5-*tert*-Butyl-2-ethyl-1-oxido-3,4-dihydro-*2H*-pyrrol-2-yl)propanoic acid (**5a**)

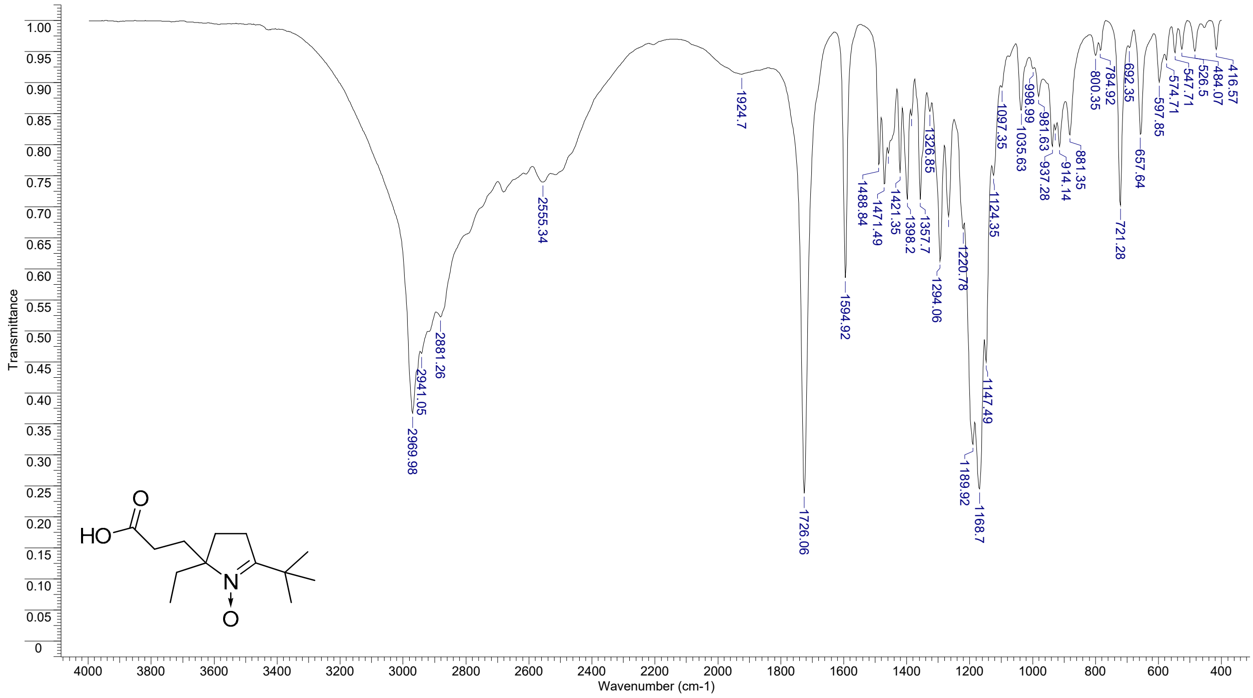

Figure S3. IR spectrum of **5a** (KBr)

1.4 3,3'-(5-*tert*-Butyl-1-oxido-3,4-dihydro-*2H*-pyrrole-2,2-diyl)dipropanoic acid (**5b**)

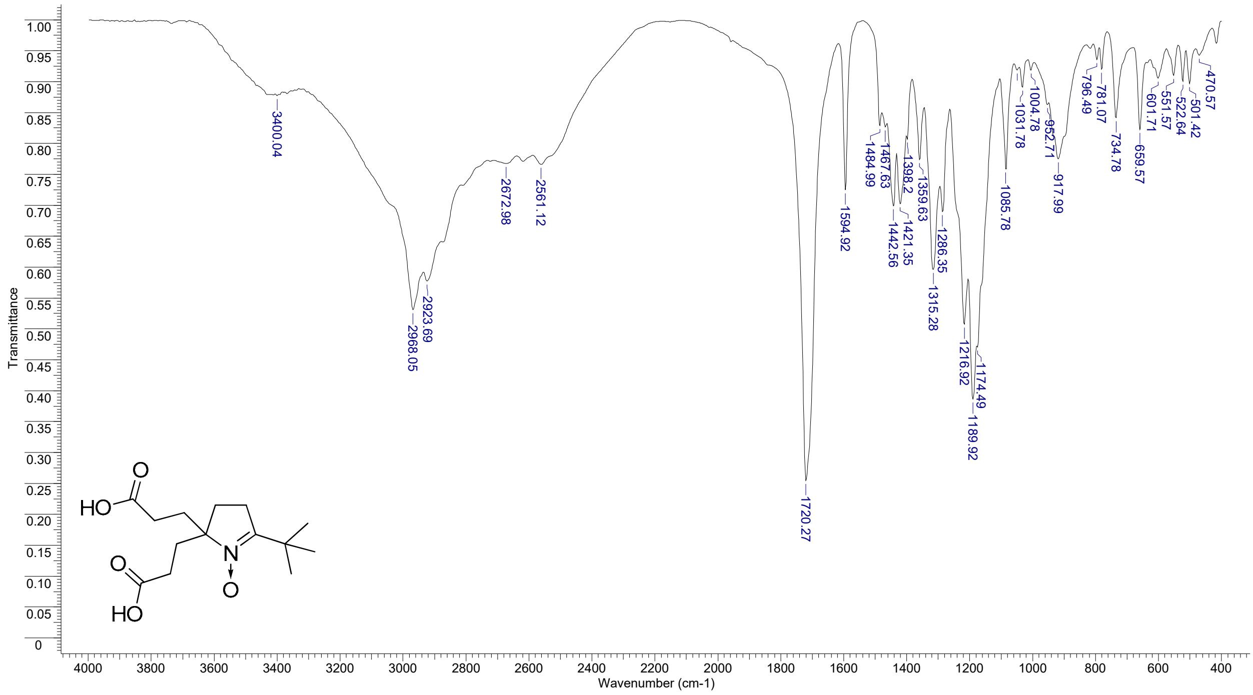

Figure S4. IR spectrum of **5b** (KBr)

1.5 2-*tert*-Butyl-5-(2-carboxyethyl)-5-ethyl-2-ethynylpyrrolidine-1-oxyl (**7a,a'**)

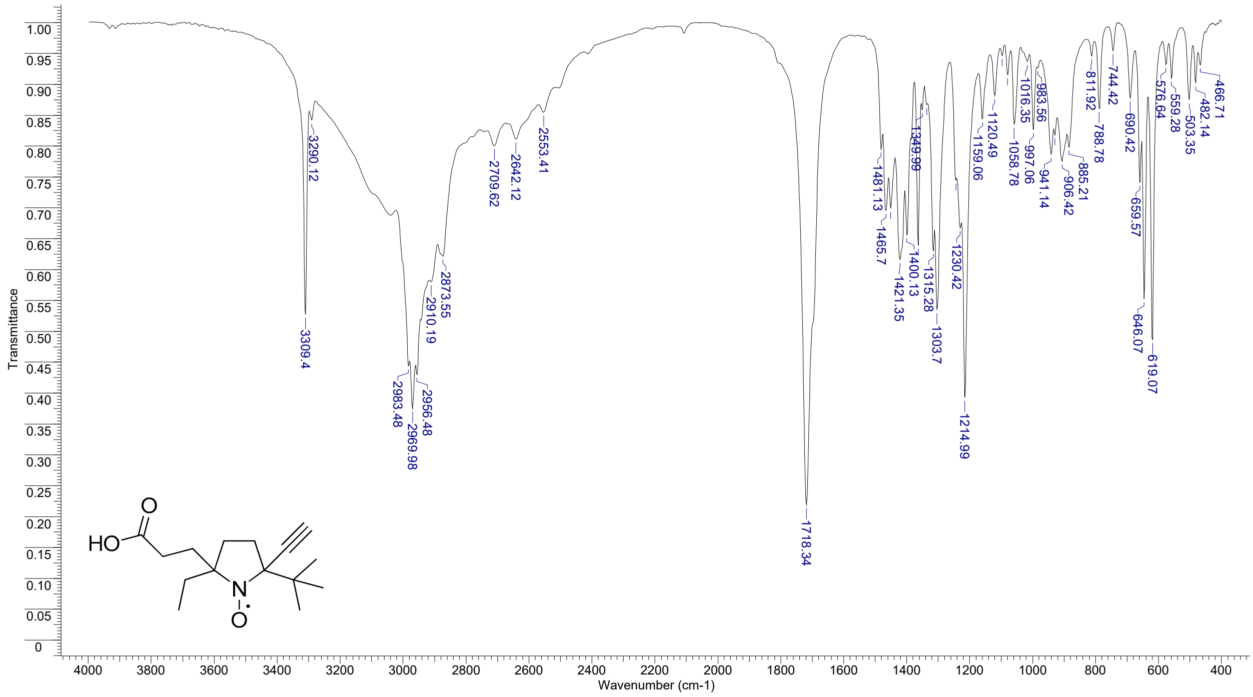

Figure S5. IR spectrum of **7a,a'**

1.6 2-*tert*-Butyl-5-ethyl-2-ethynyl-5-(3-methoxy-3-oxopropyl)pyrrolidine-1-oxyl (**8a**)

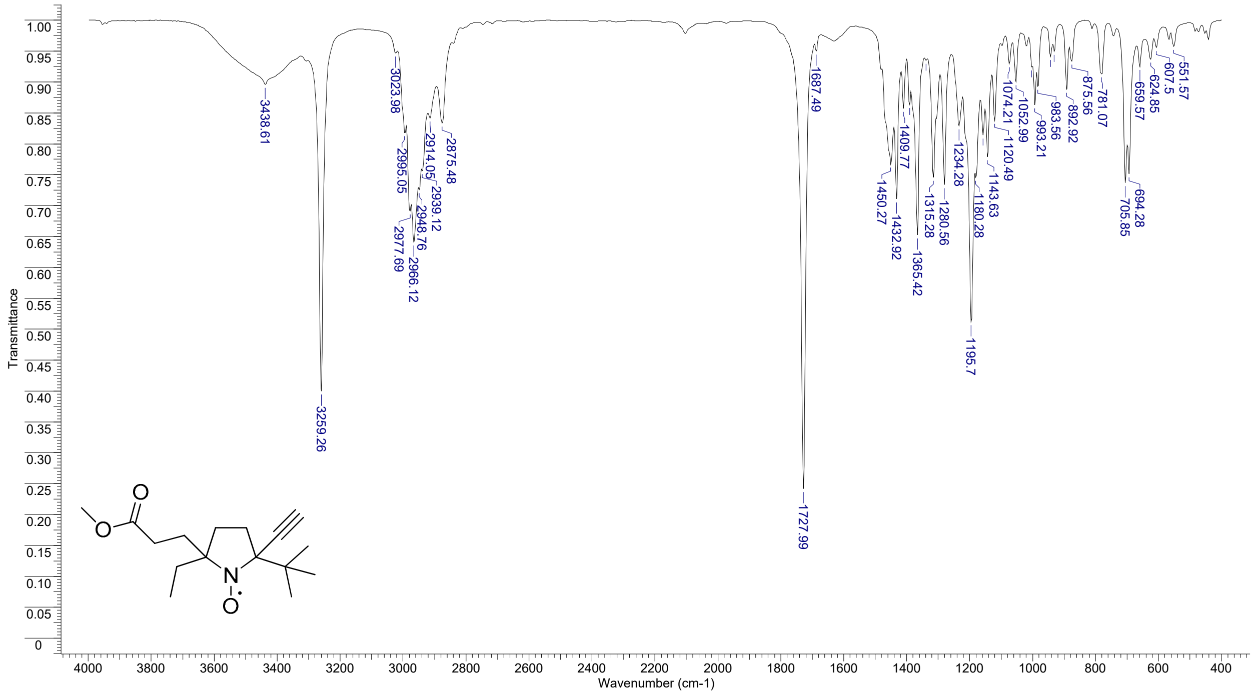

Figure S6. IR spectrum of **8a** (KBr)

1.7 2-*tert*-Butyl-5-(2-carboxyethyl)-2,5-diethylpyrrolidine-1-oxyl (**10a**)

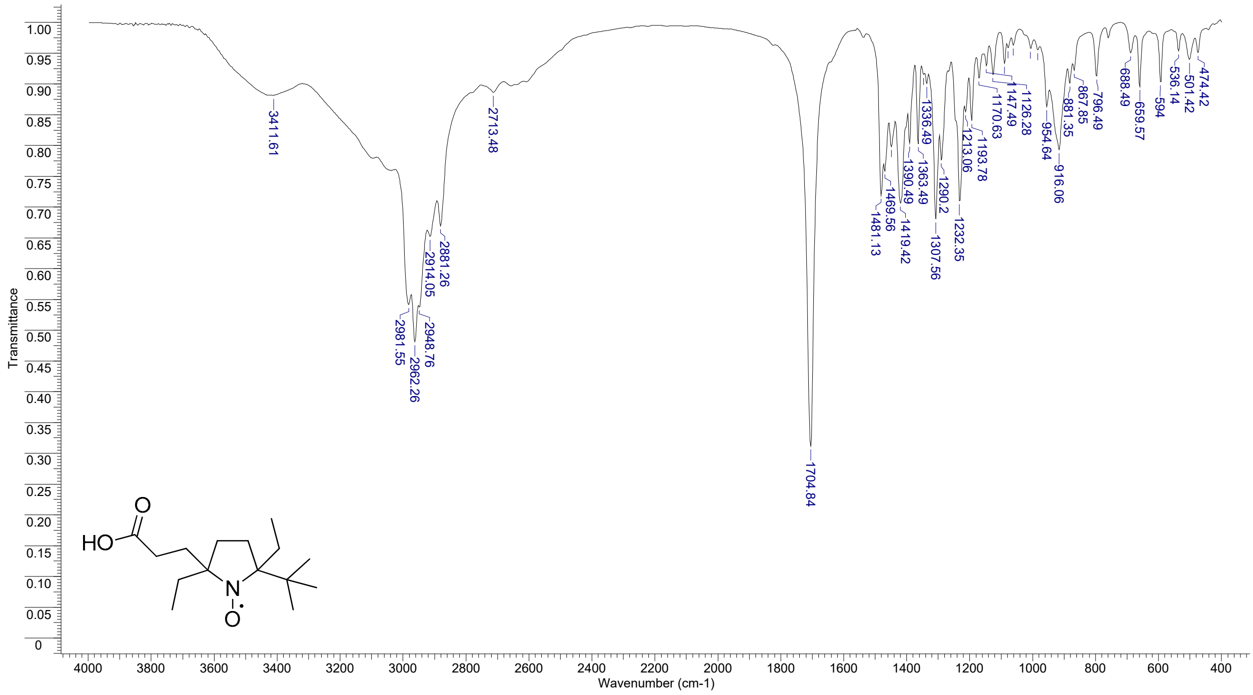

Figure S7. IR spectrum of **10a** (KBr)

1.8 3-(5-*tert*-Butyl-2-ethyl-1-oxido-3,4-dihydro-*2H*-pyrrol-2-yl)-1-propanol (**11a**)

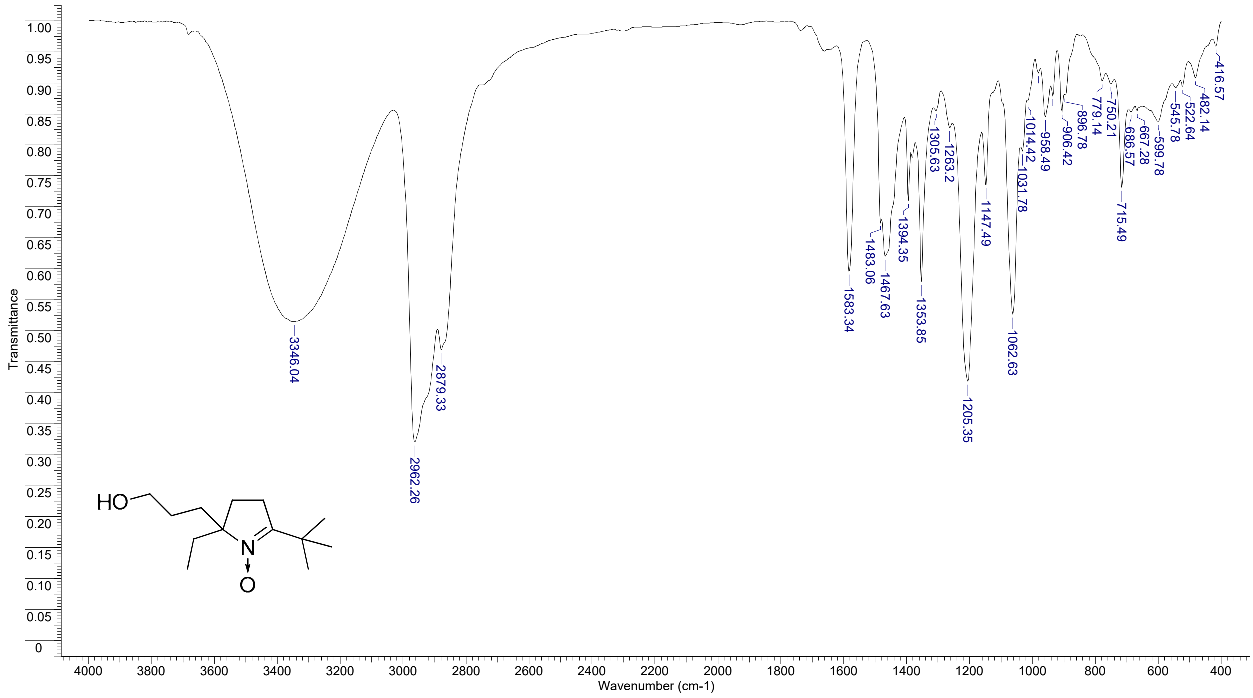

Figure S8. IR spectrum of **11a** (neat)

1.9 3,3'-(5-*tert*-Butyl-1-oxido-3,4-dihydro-*2H*-pyrrole-2,2-diyl)di(1-propanol) (**11b**)

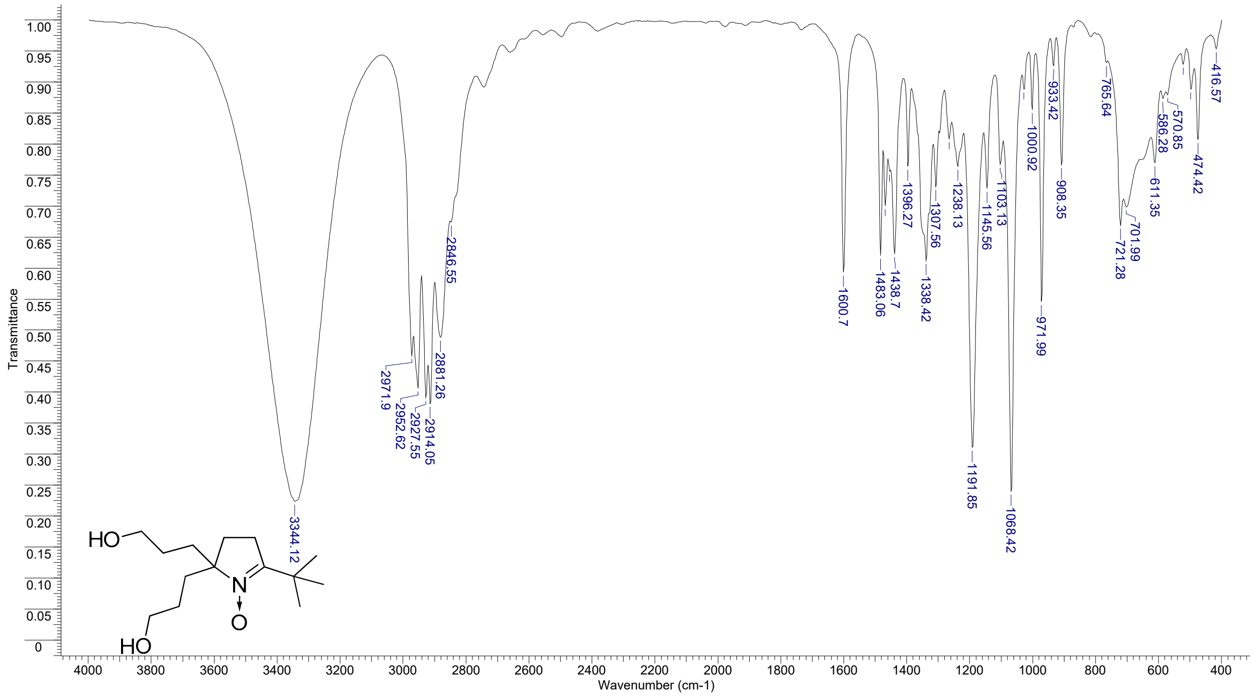

Figure S9. IR spectrum of **11b** (KBr)

1.10 5-*tert*-Butyl-2-ethyl-2-[3-(1-methoxy-1-methylethoxy)propyl]-3,4-dihydro-*2H*-pyrrole 1-oxide (**12a**)

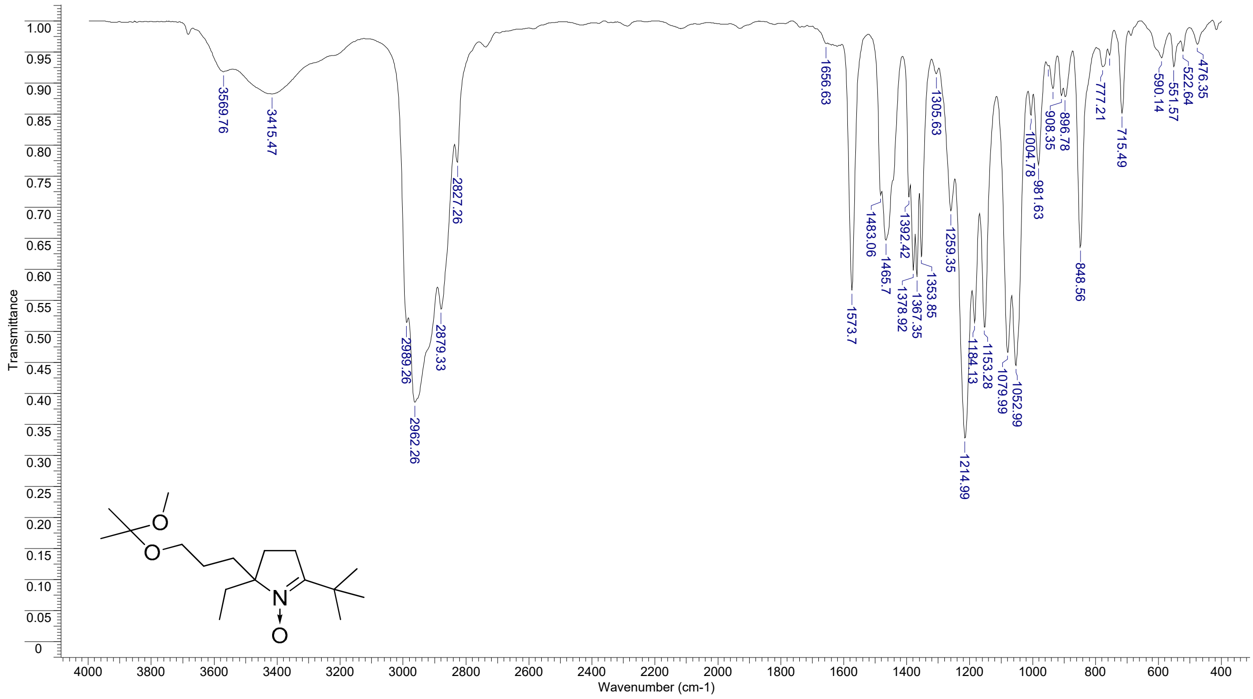

Figure S10. IR spectrum of **12a** (neat)

1.11 5-*tert*-Butyl-2,2-bis[3-(1-methoxy-1-methylethoxy)propyl]-3,4-dihydro-*2H*-pyrrole 1-oxide (**12b**)

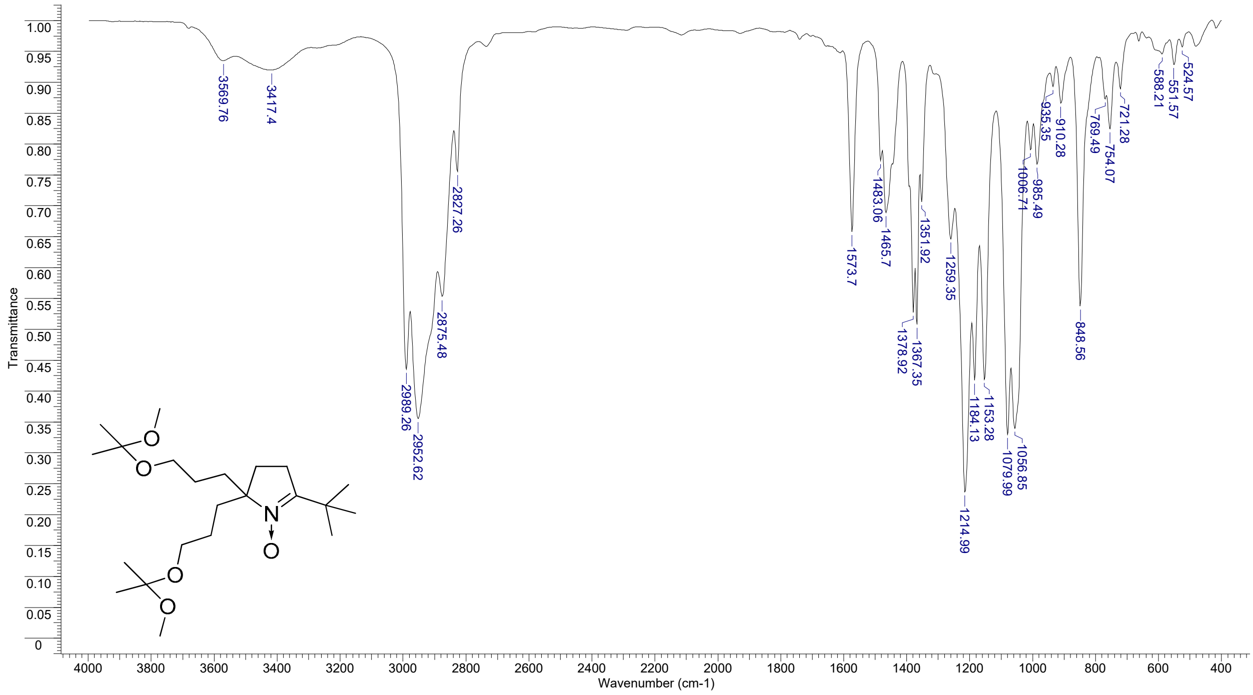

Figure S11. IR spectrum of **12b** (neat)

1.12 2-*tert*-Butyl-2-ethyl-5,5-bis(3-hydroxypropyl)pyrrolidine-1-oxyl (**14a**)

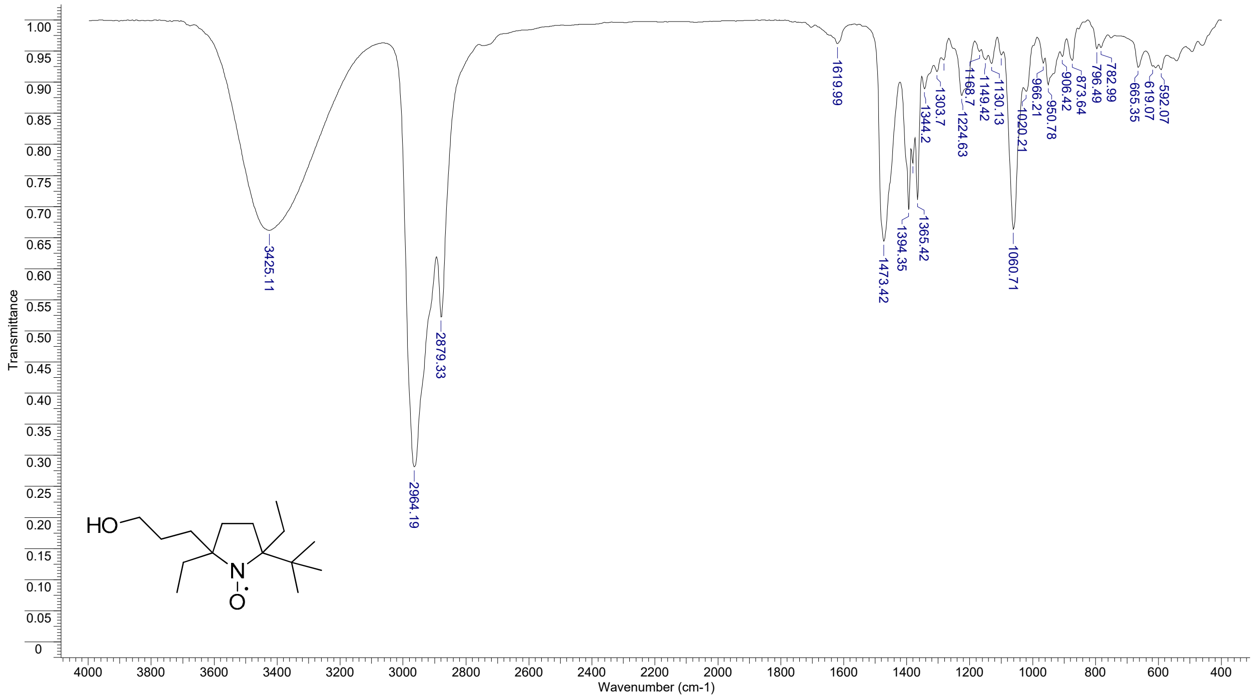

Figure S12. IR spectrum of **14a** (neat)

1.13 2-*tert*-Butyl-2-ethyl-5,5-bis(3-hydroxypropyl)pyrrolidine-1-oxyl (**14b**)

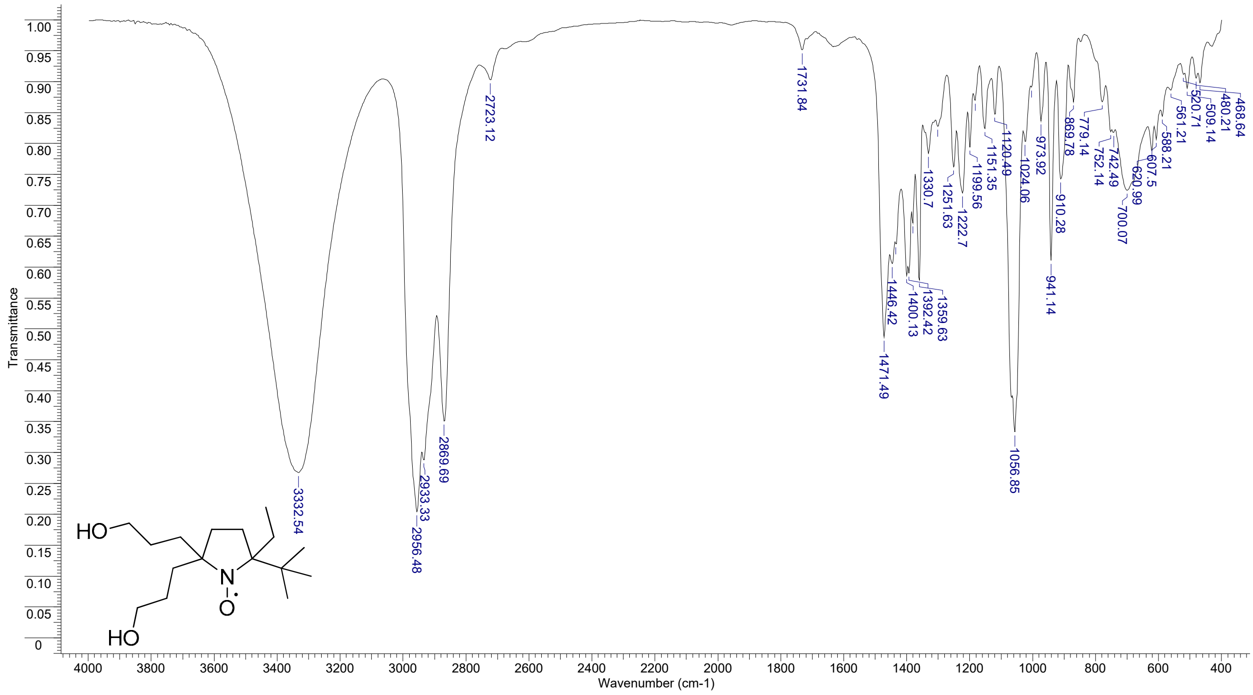

Figure S13. IR spectrum of **14b** (KBr)

2. ^{1}H and ^{13}C NMR spectral data

2.1 Methyl 3-(5-*tert*-butyl-2-ethyl-1-oxido-3,4-dihydro-*2H*-pyrrol-2-yl)propanoate (**4a**)

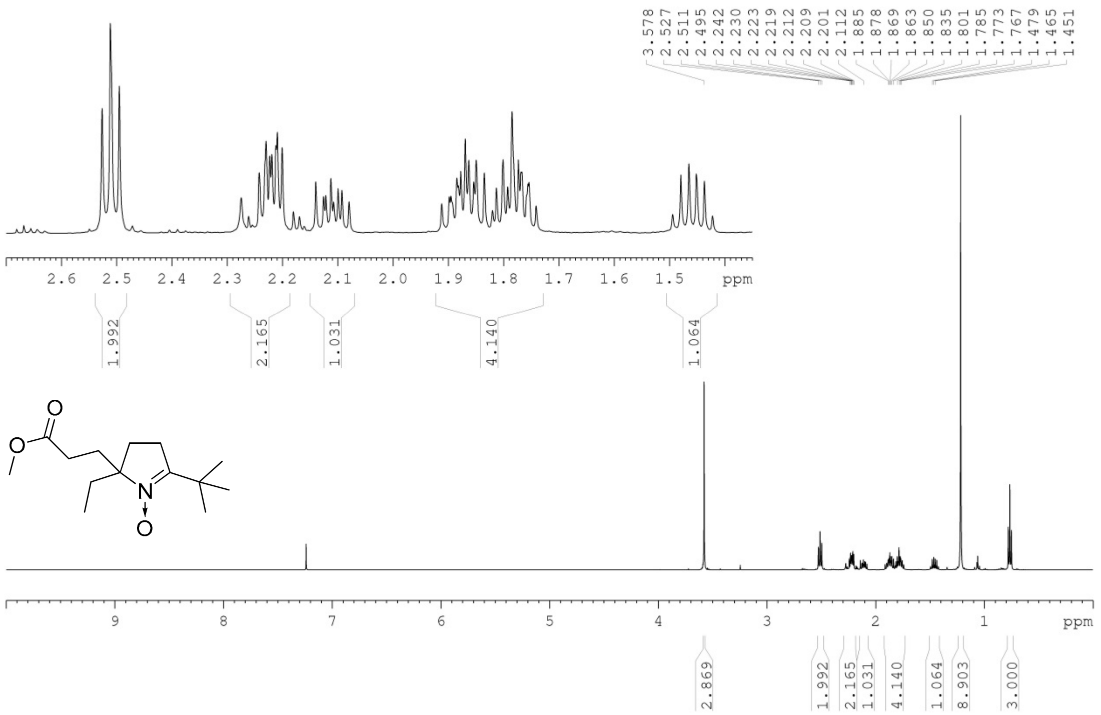

Figure S14. ^{1}H NMR spectrum of **4a** in $CDCl_3$ at 500 MHz

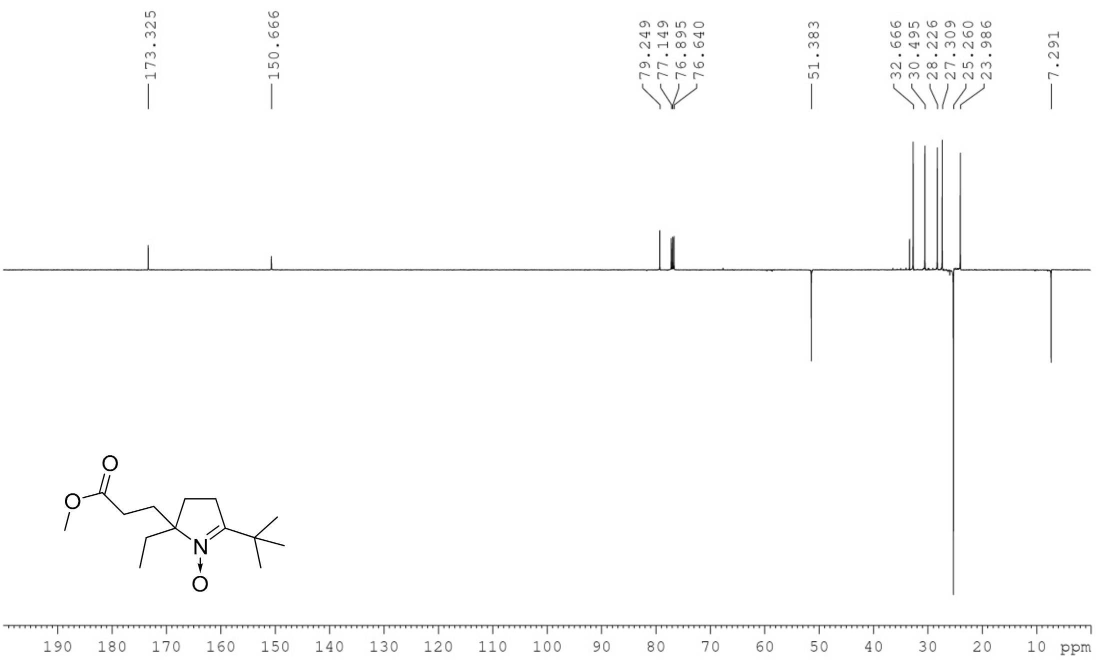

Figure S15. ^{13}C NMR spectrum of **4a** in $CDCl_3$ at 125 MHz

2.2 Dimethyl 3,3'-(5-*tert*-butyl-1-oxido-3,4-dihydro-*2H*-pyrrole-2,2-diyl)dipropanoate (**4b**)

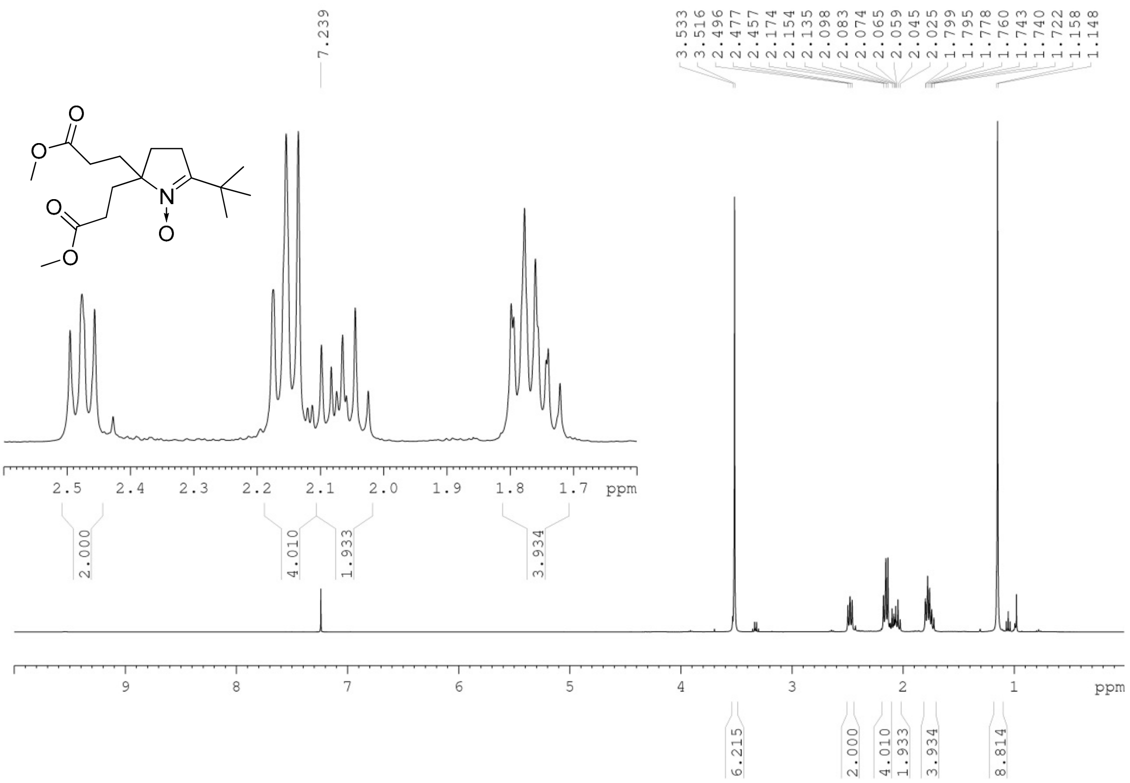

Figure S16. ^{1}H NMR spectrum of **4b** in $CDCl_3$ at 400 MHz

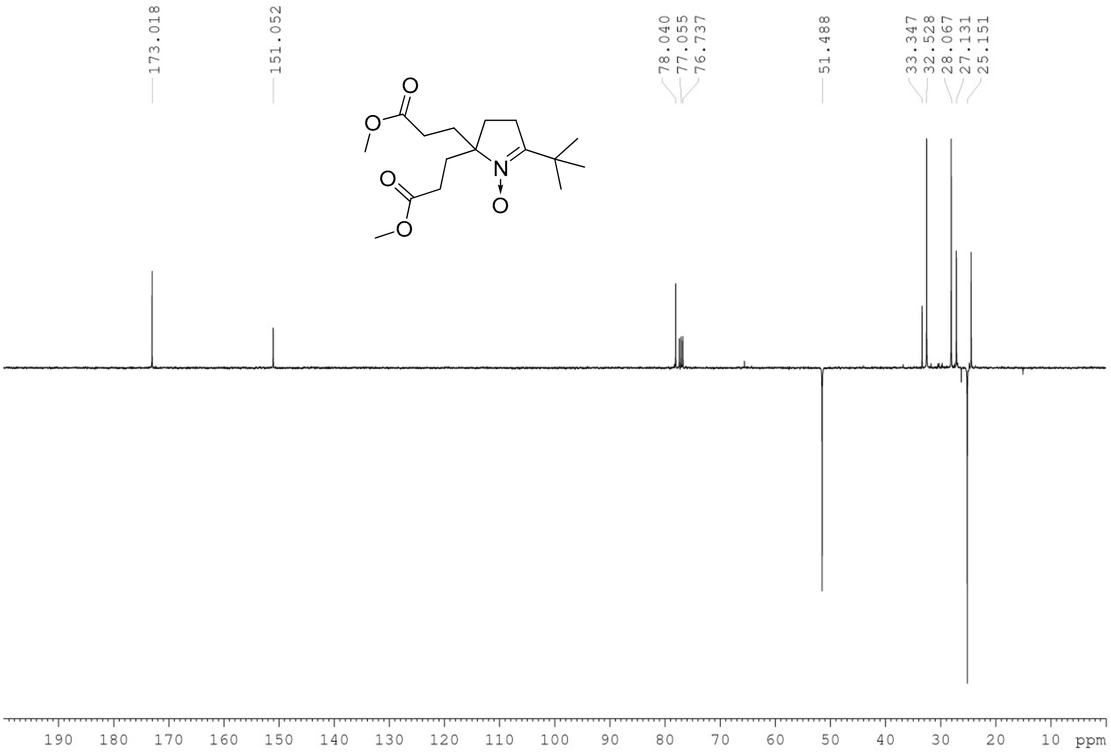

Figure S17. ^{13}C NMR spectrum of **4b** in $CDCl_3$ at 100 MHz

2.3 3-(5-*tert*-Butyl-2-ethyl-1-oxido-3,4-dihydro-*2H*-pyrrol-2-yl)propanoic acid (**5a**)

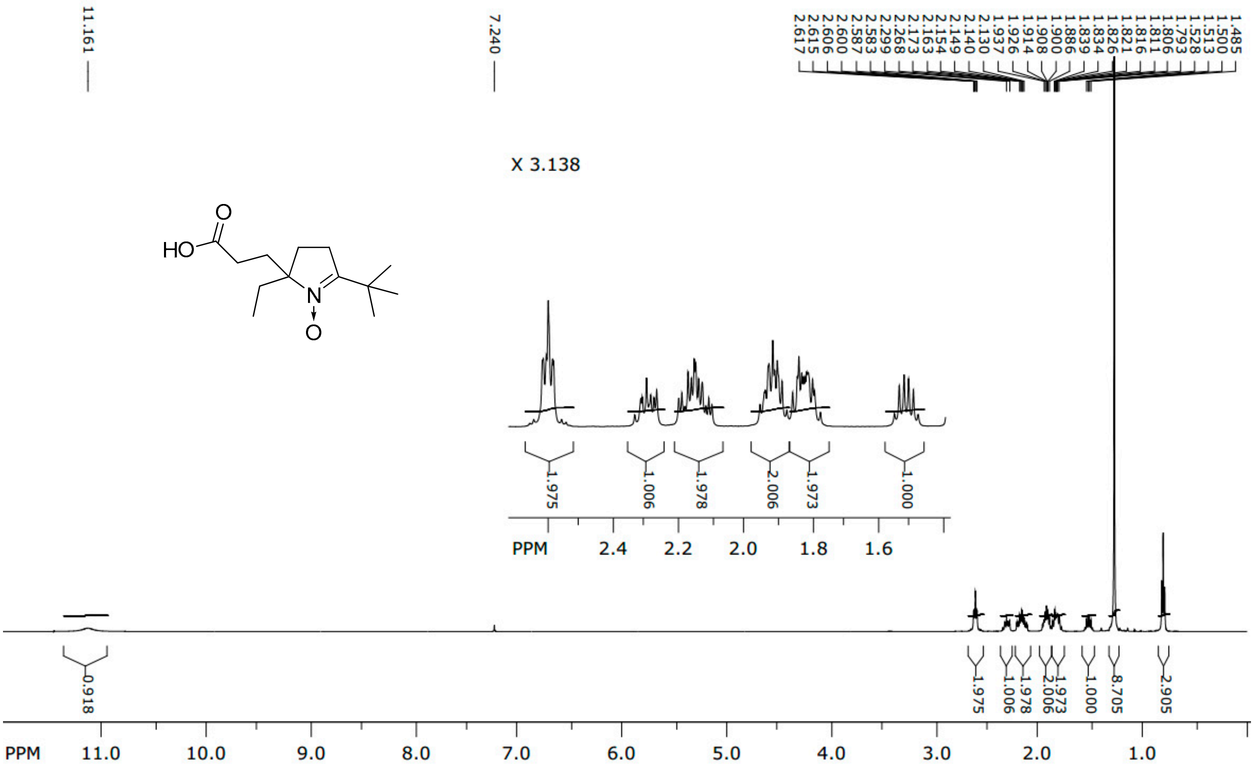

Figure S18. ^{1}H NMR spectrum of **5a** in $CDCl_3$ at 500 MHz

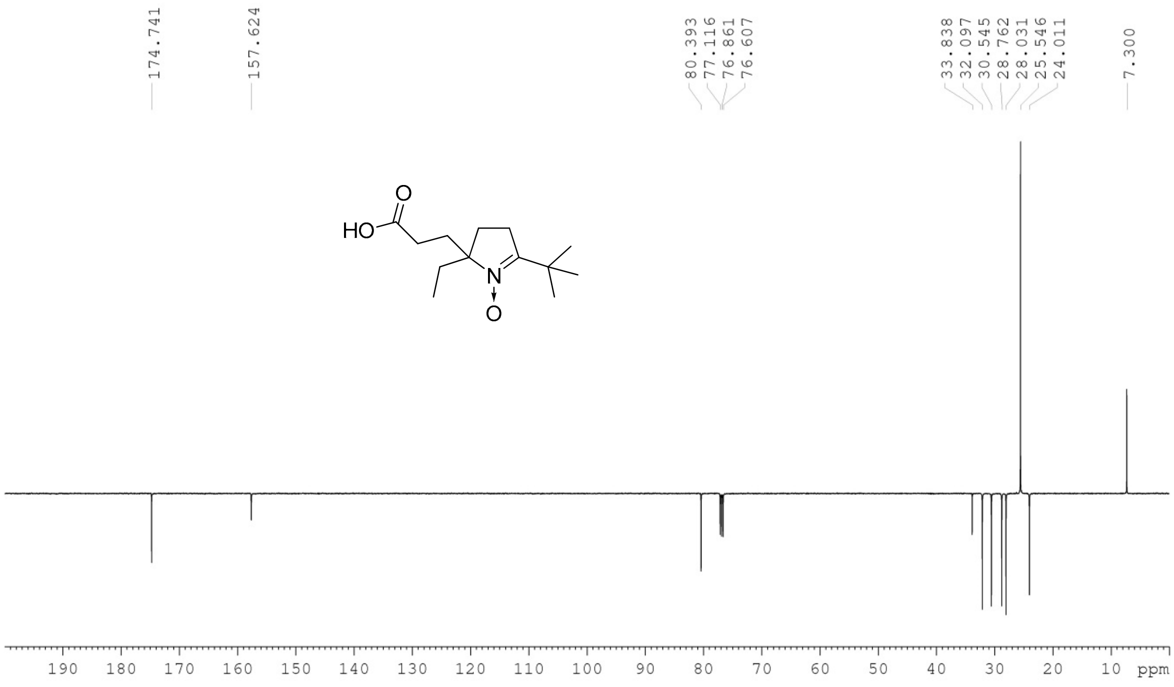

Figure S19. ^{13}C NMR spectrum of **5a** in $CDCl_3$ at 125 MHz

2.4 3,3'-(5-*tert*-Butyl-1-oxido-3,4-dihydro-*2H*-pyrrole-2,2-diyl)dipropanoic acid (**5b**)

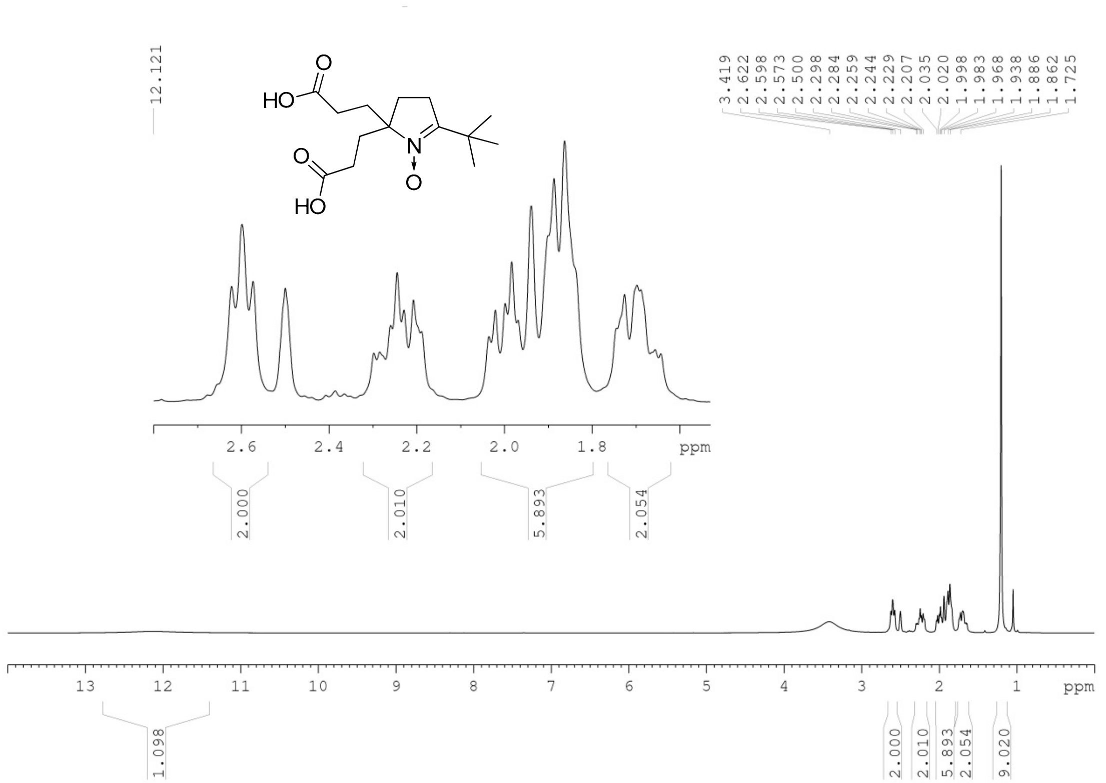

Figure S20. ^{1}H NMR spectrum of **5b** in DMSO-d^{6} at 300 MHz

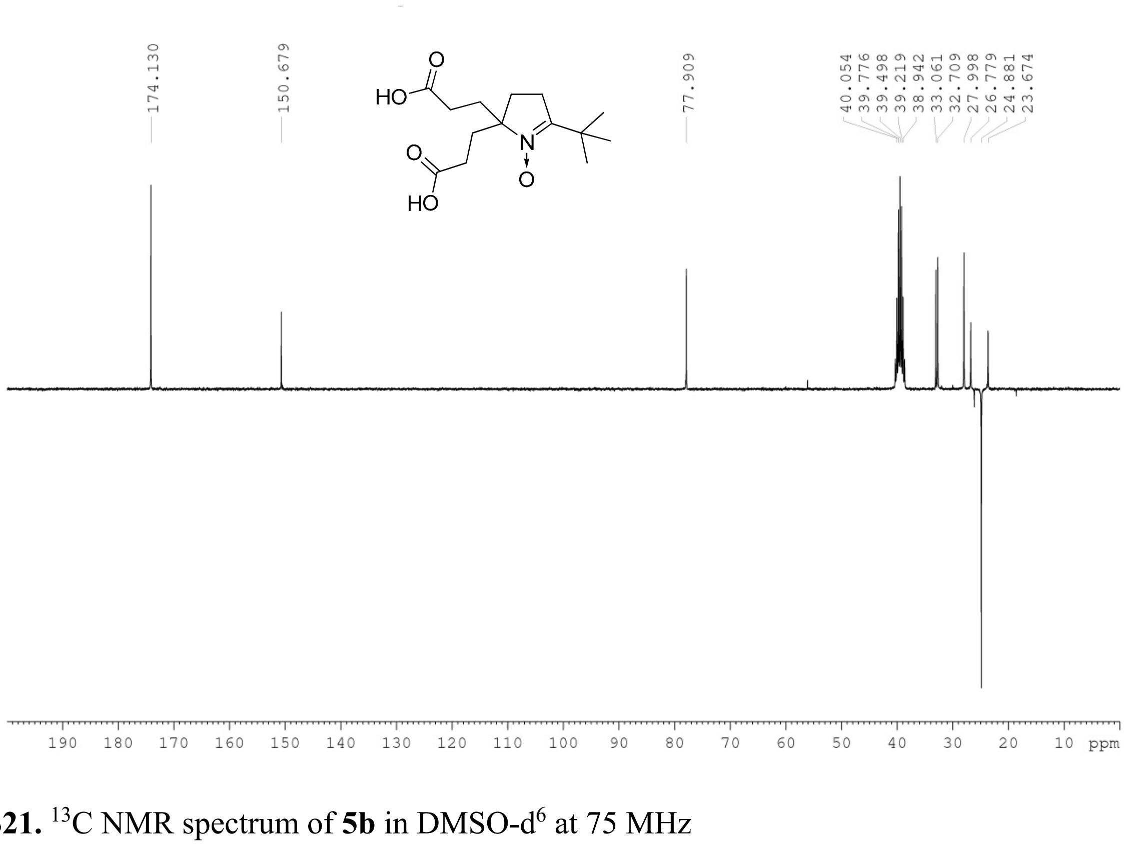

Figure S21. ^{13}C NMR spectrum of **5b** in DMSO-d^{6} at 75 MHz

2.5 2-*tert*-Butyl-5-(2-carboxyethyl)-5-ethyl-2-ethynylpyrrolidinium trifluoroacetate (mixture of diastereomers) (**7a,a'_*red***)

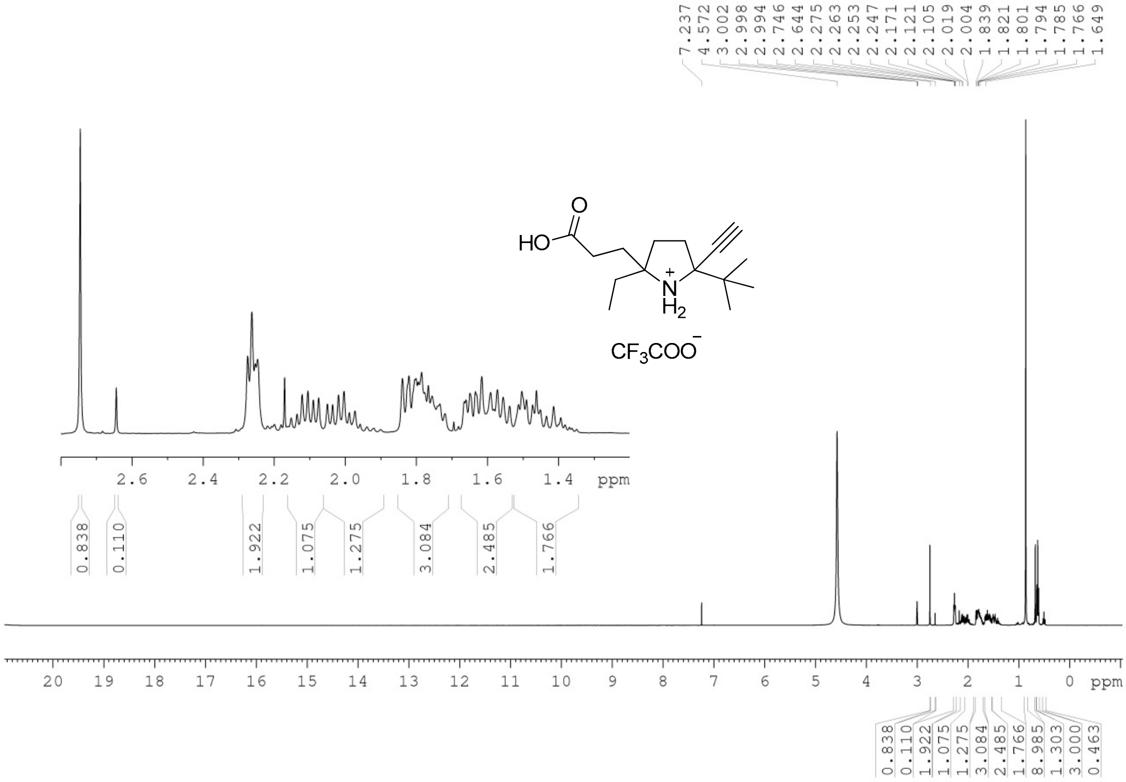

Figure S22. ^{1}H NMR spectrum of **7a,a'_*red*** in $CDCl_3$-CD_3OD at 400 MHz

2.6 2-*tert*-Butyl-5-ethyl-2-ethynyl-5-(3-methoxy-3-oxopropyl)pyrrolidinium trifluoroacetate (**8a_red**)

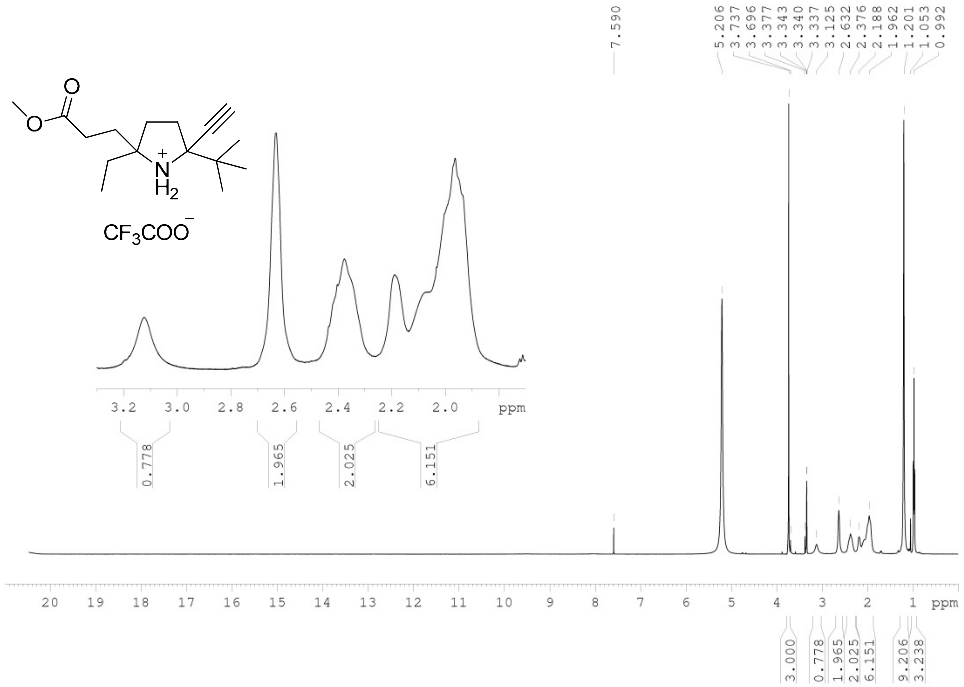

Figure S23. ^{1}H NMR spectrum of **8a_*red*** in $CDCl_3$-CD_3OD at 500 MHz at 298 K

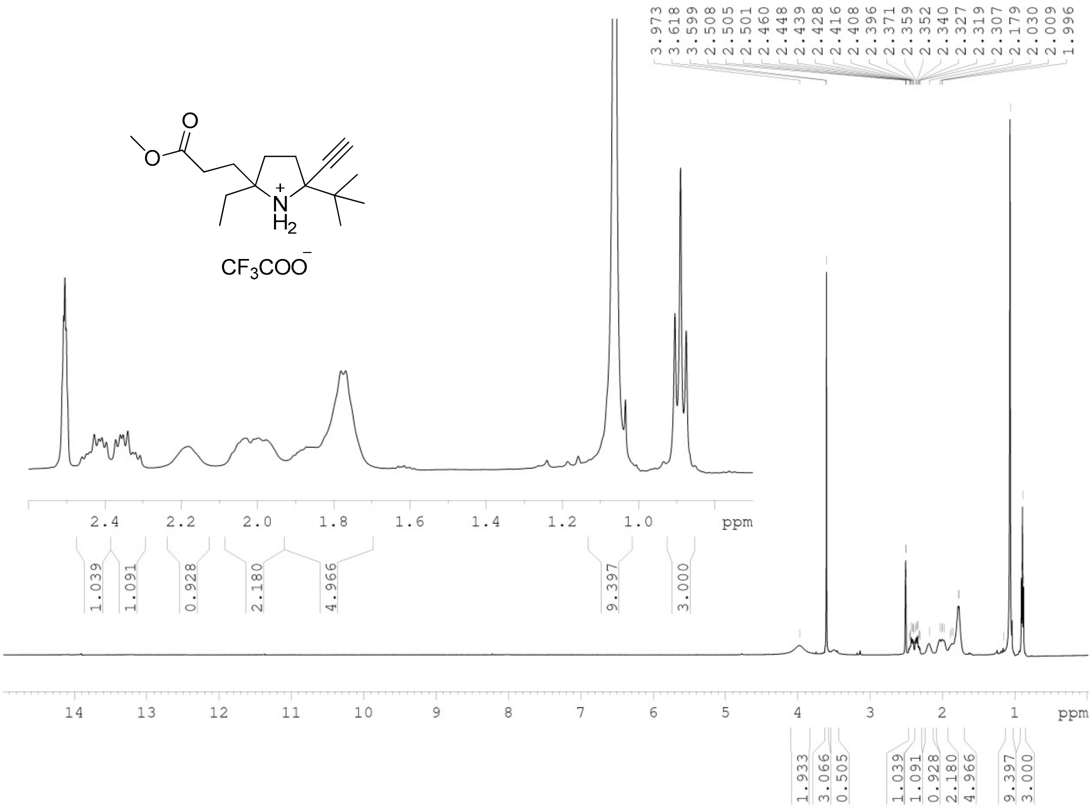

Figure S24. ^{1}H NMR spectrum of **8a_*red*** in DMSO-d^{6} at 500 MHz at 333 K

2.7 2-*tert*-Butyl-5-(2-carboxyethyl)-2,5-diethylpyrrolidinium trifluoroacetate (**10a_*red***)

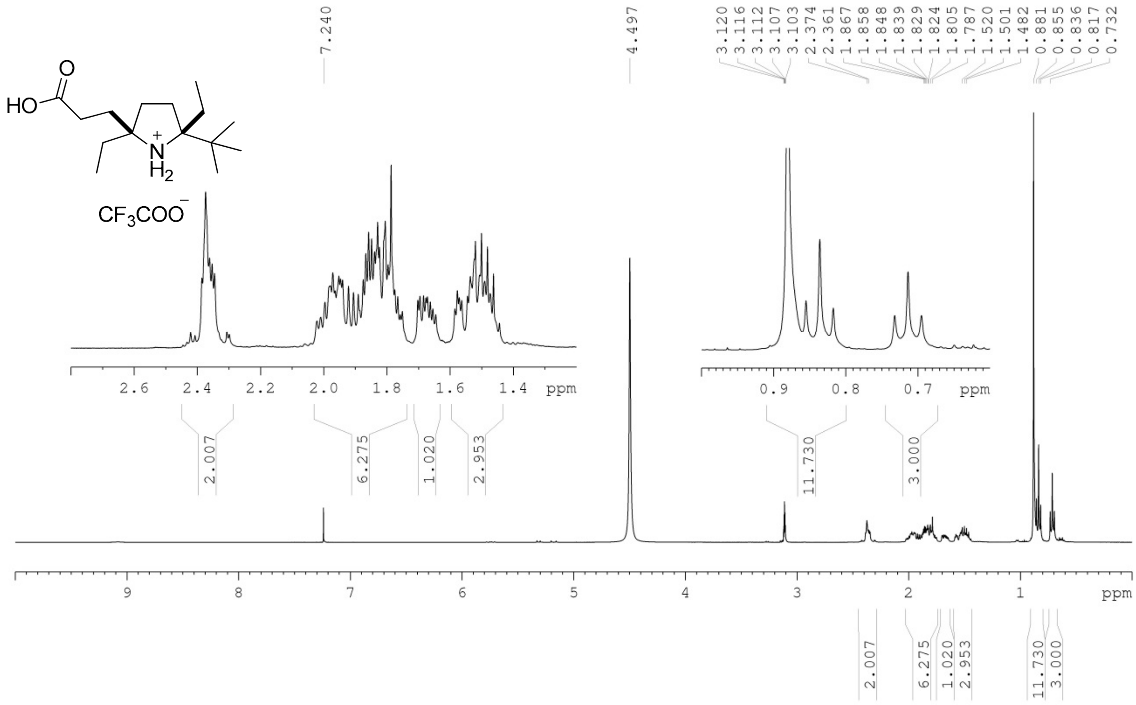

Figure S25. ^{1}H NMR spectrum of **10a_*red*** in $CDCl_3$-CD_3OD at 400 MHz

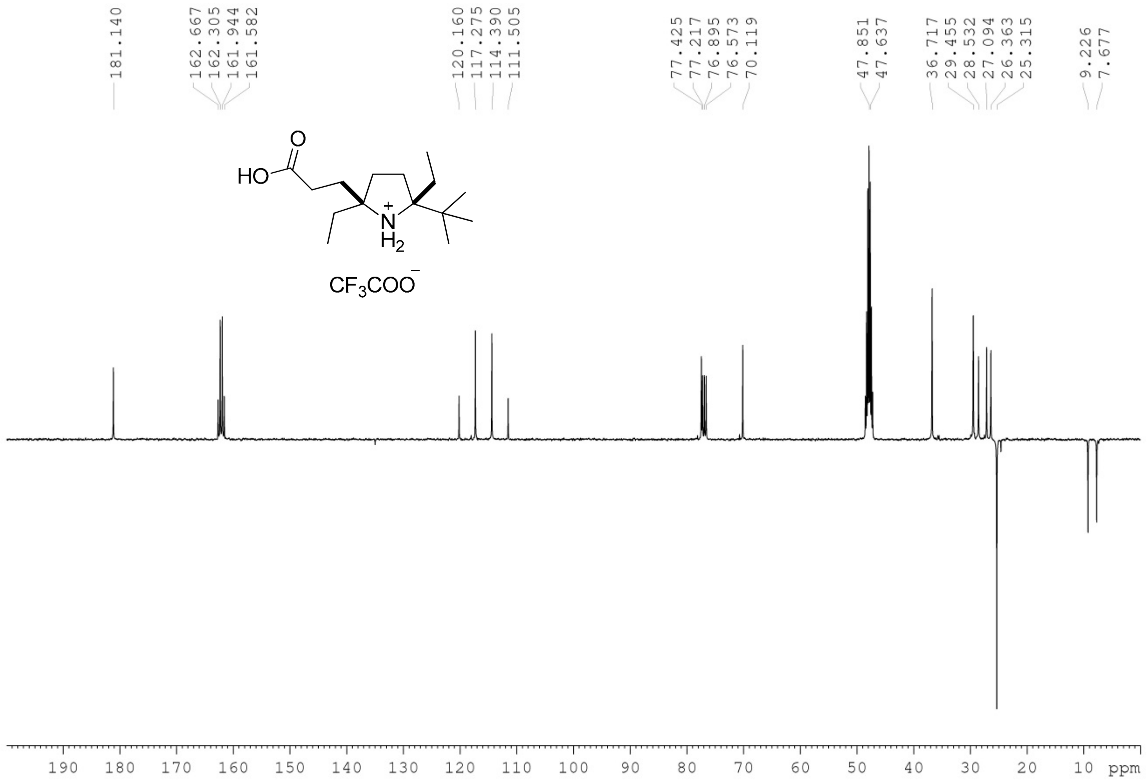

Figure S26. ^{13}C NMR spectrum of **10a_*red*** in $CDCl_3$-CD_3OD at 100 MHz

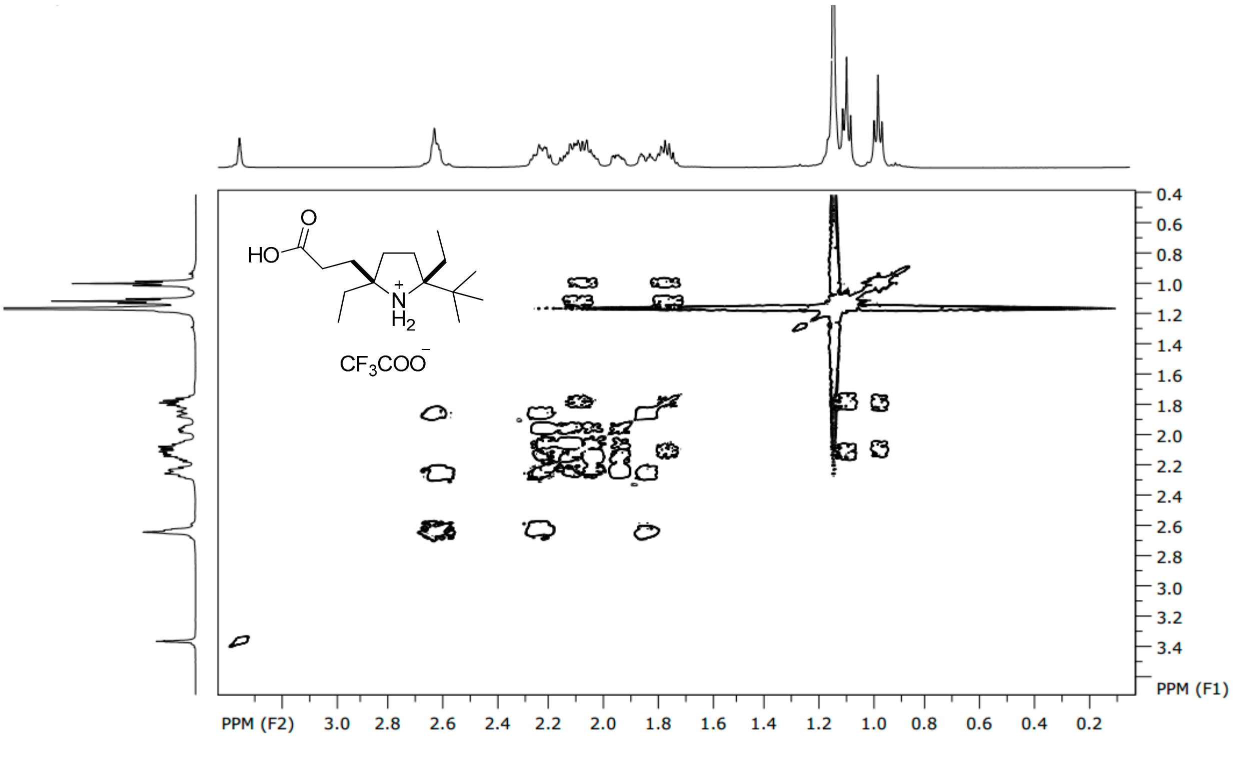

Figure S27. ^{1}H-^{1}H COSY NMR spectrum of **10a_*red*** in $CDCl_3$-CD_3OD

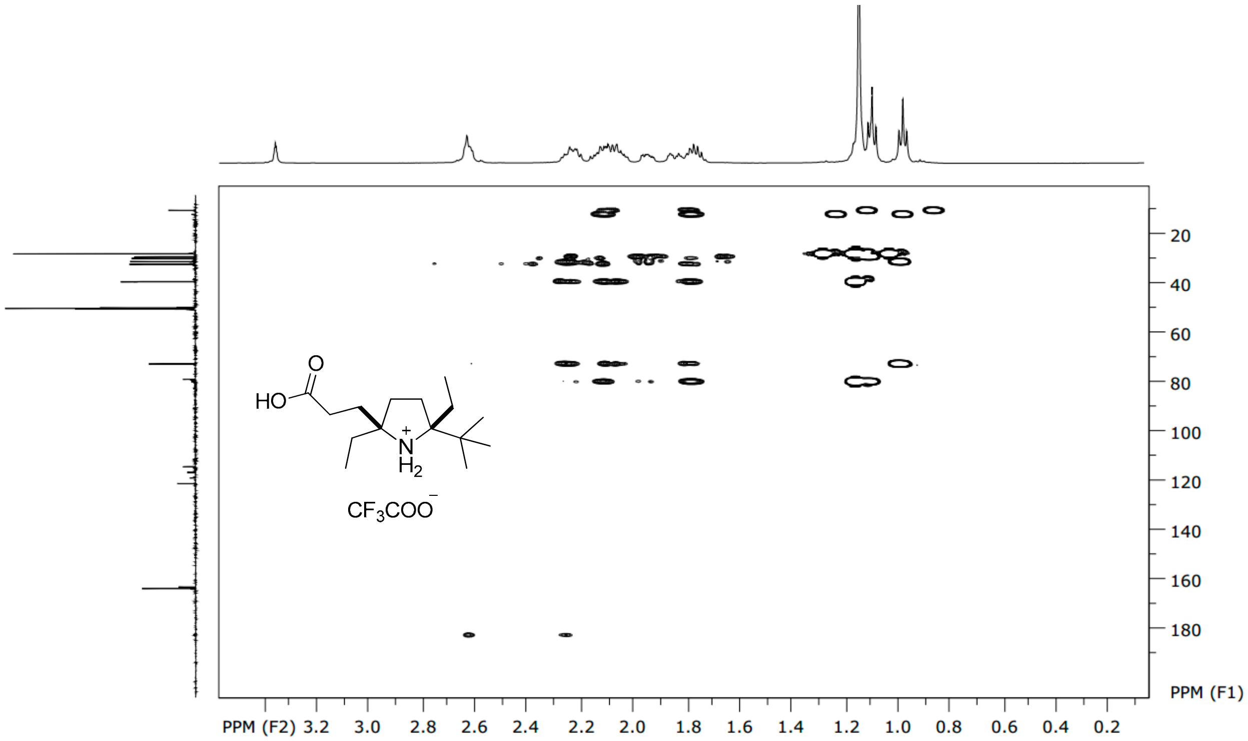

Figure S28. ^{1}H-^{13}C HMBC NMR spectrum of **10a_*red*** in CDCl3-CD3OD

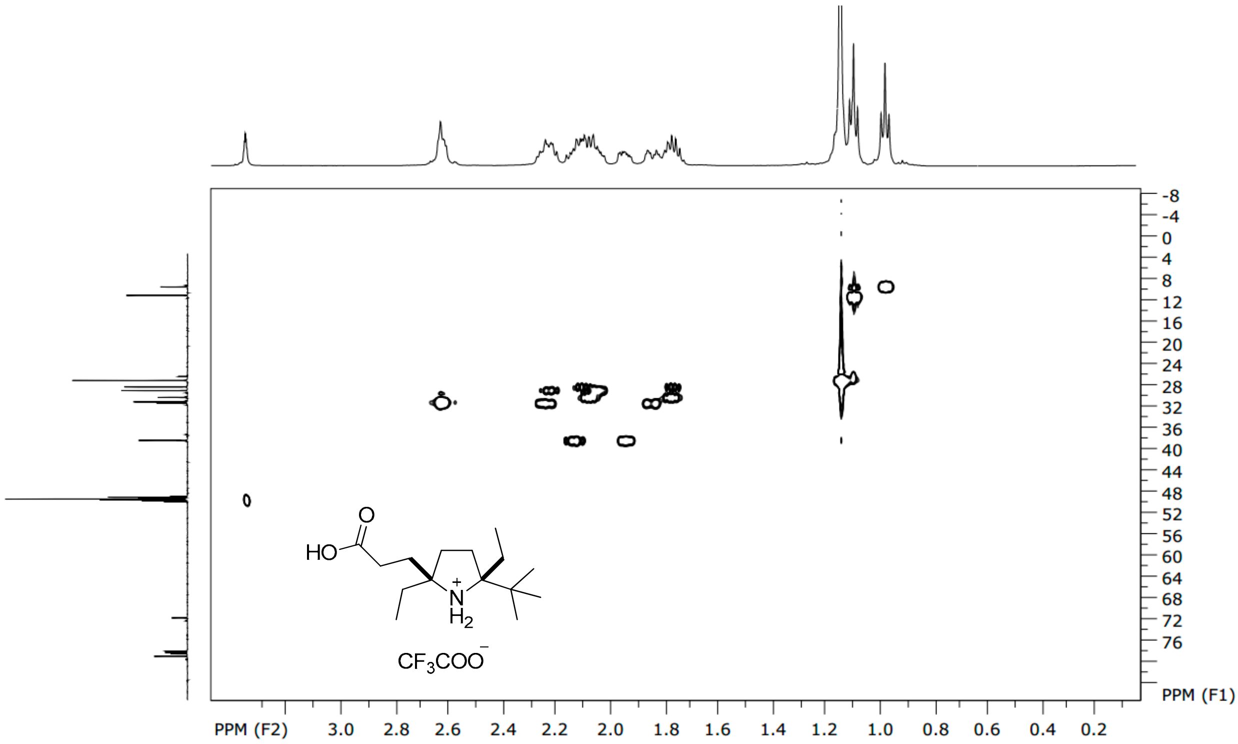

Figure S29. ^{1}H-^{13}C HSQC NMR spectrum of **10a_*red*** in CDCl3-CD3OD

2.8 3-(5-*tert*-Butyl-2-ethyl-1-oxido-3,4-dihydro-*2H*-pyrrol-2-yl)-1-propanol (**11a**)

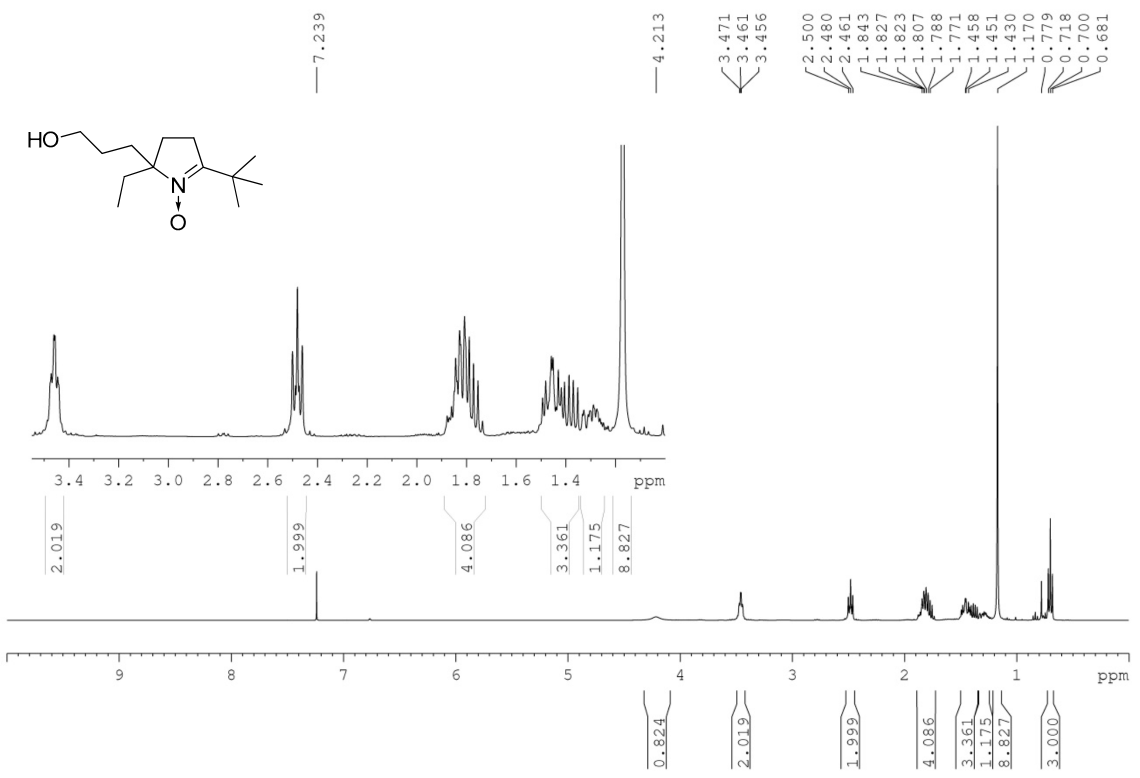

Figure S30. ^{1}H NMR spectrum of **11a** in $CDCl_3$ at 400 MHz

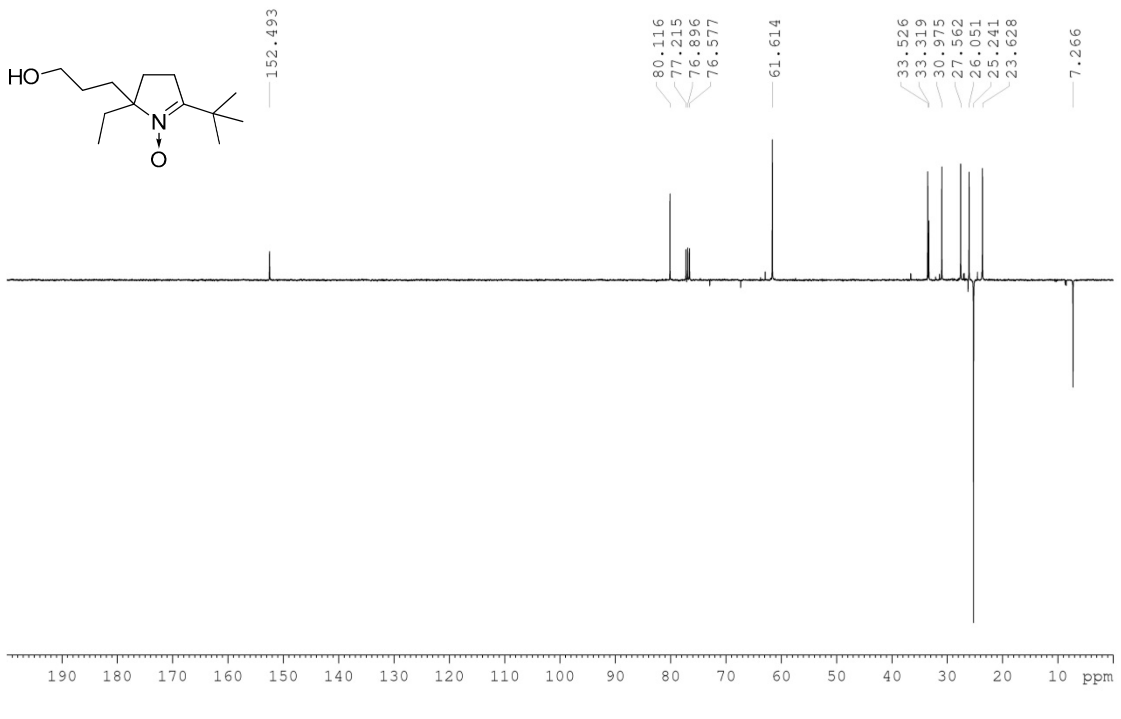

Figure S31. ^{13}C NMR spectrum of **11a** in $CDCl_3$ at 100 MHz

2.9 3,3'-(5-*tert*-Butyl-1-oxido-3,4-dihydro-*2H*-pyrrole-2,2-diyl)di(1-propanol) (**11b**)

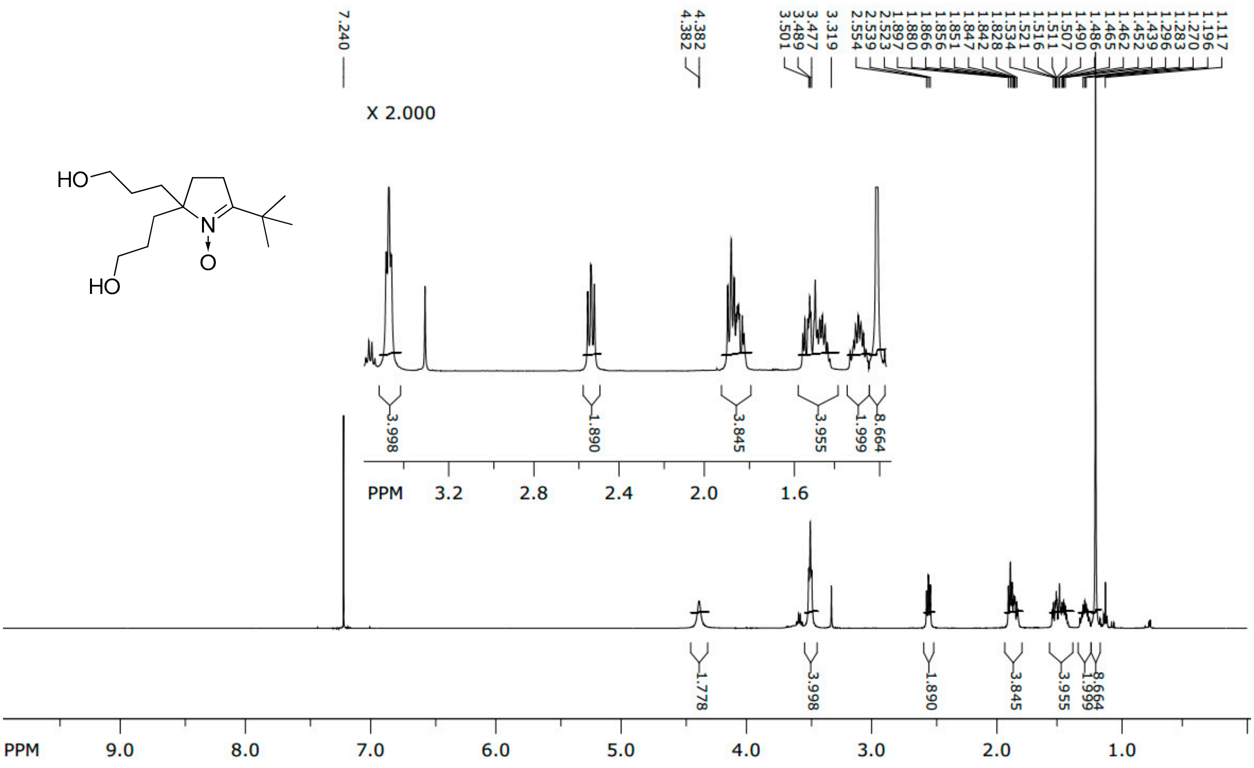

Figure S32. ^{1}H NMR spectrum of **11b** in $CDCl_3$ at 500 MHz

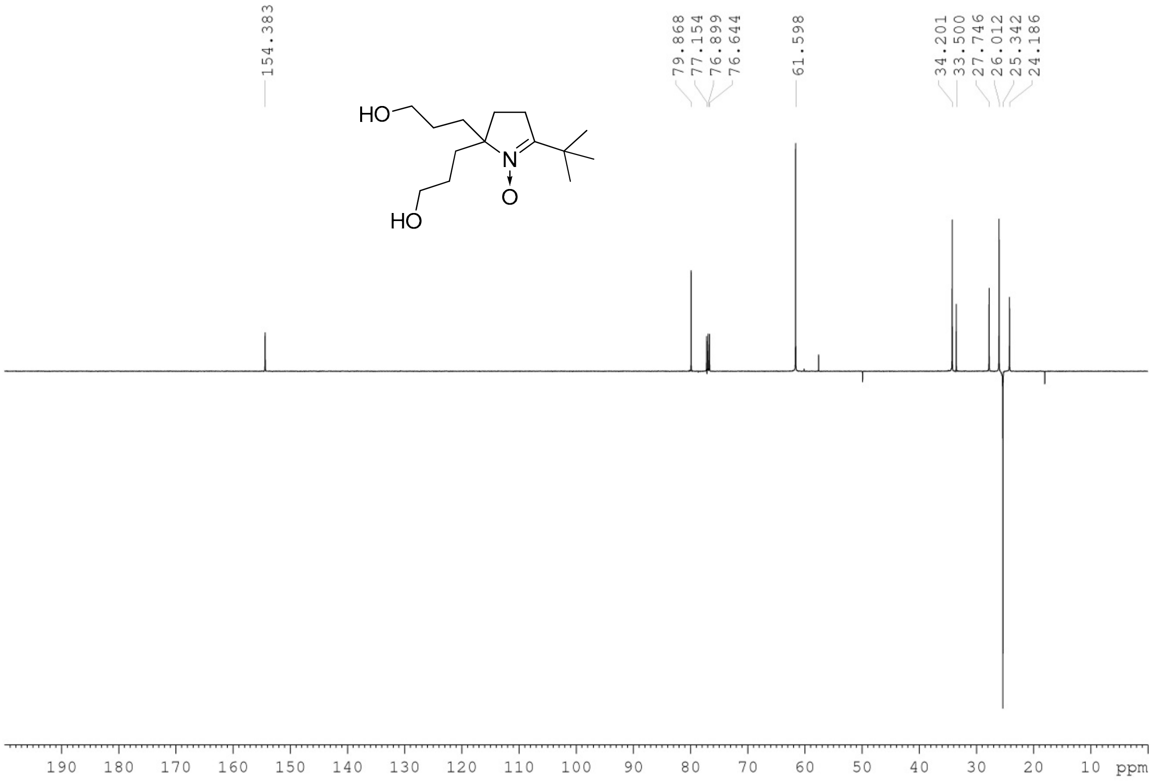

Figure S33. ^{13}C NMR spectrum of **11b** in $CDCl_3$ at 125 MHz

2.10 5-*tert*-Butyl-2-ethyl-2-[3-(1-methoxy-1-methylethoxy)propyl]-3,4-dihydro-*2H*-pyrrole 1-oxide (**12a**)

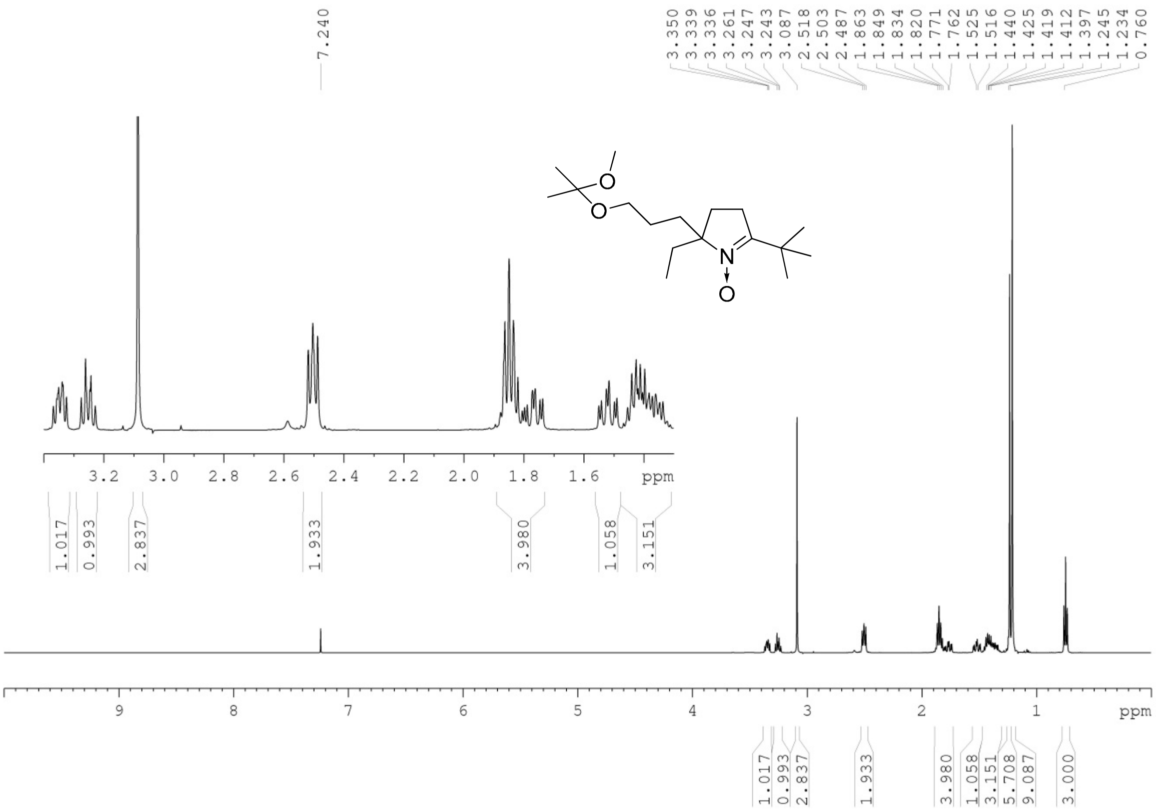

Figure S34. ^{1}H NMR spectrum of **12a** in $CDCl_3$ at 500 MHz

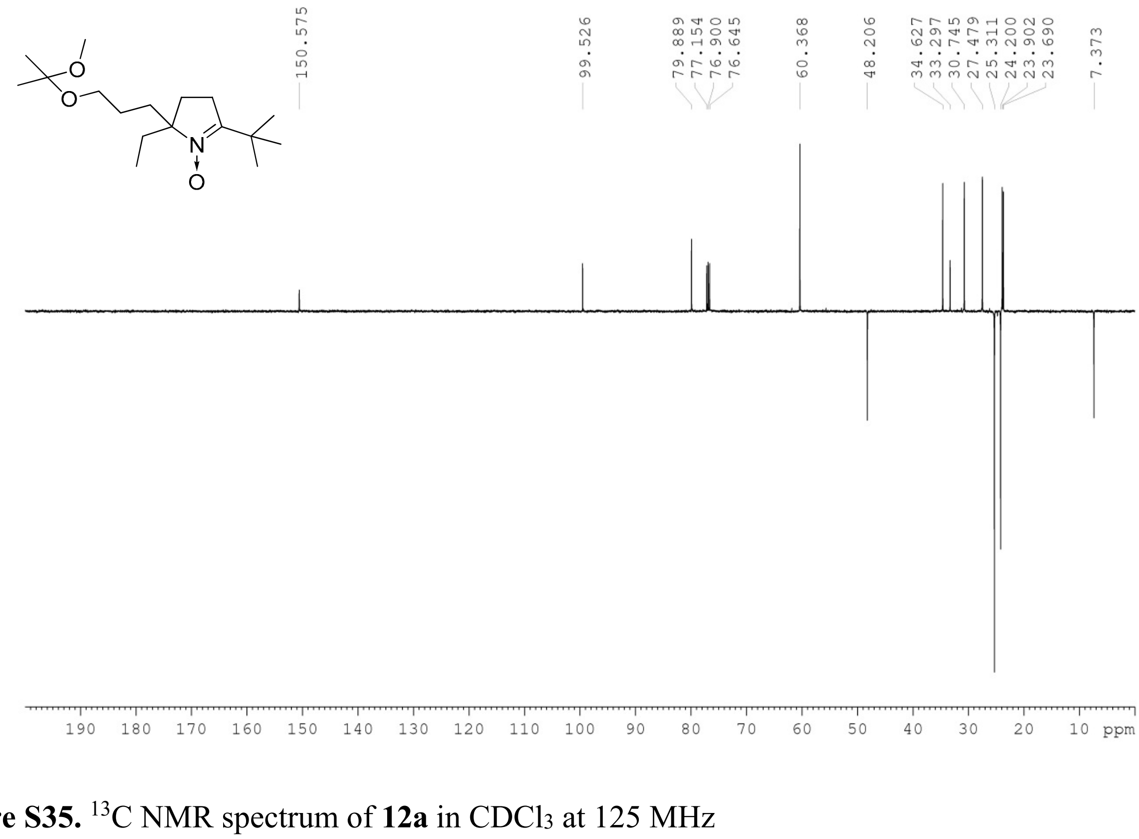

Figure S35. ^{13}C NMR spectrum of **12a** in $CDCl_3$ at 125 MHz

2.11 5-*tert*-Butyl-2,2-bis[3-(1-methoxy-1-methylethoxy)propyl]-3,4-dihydro-*2H*-pyrrole 1-oxide (**12b**)

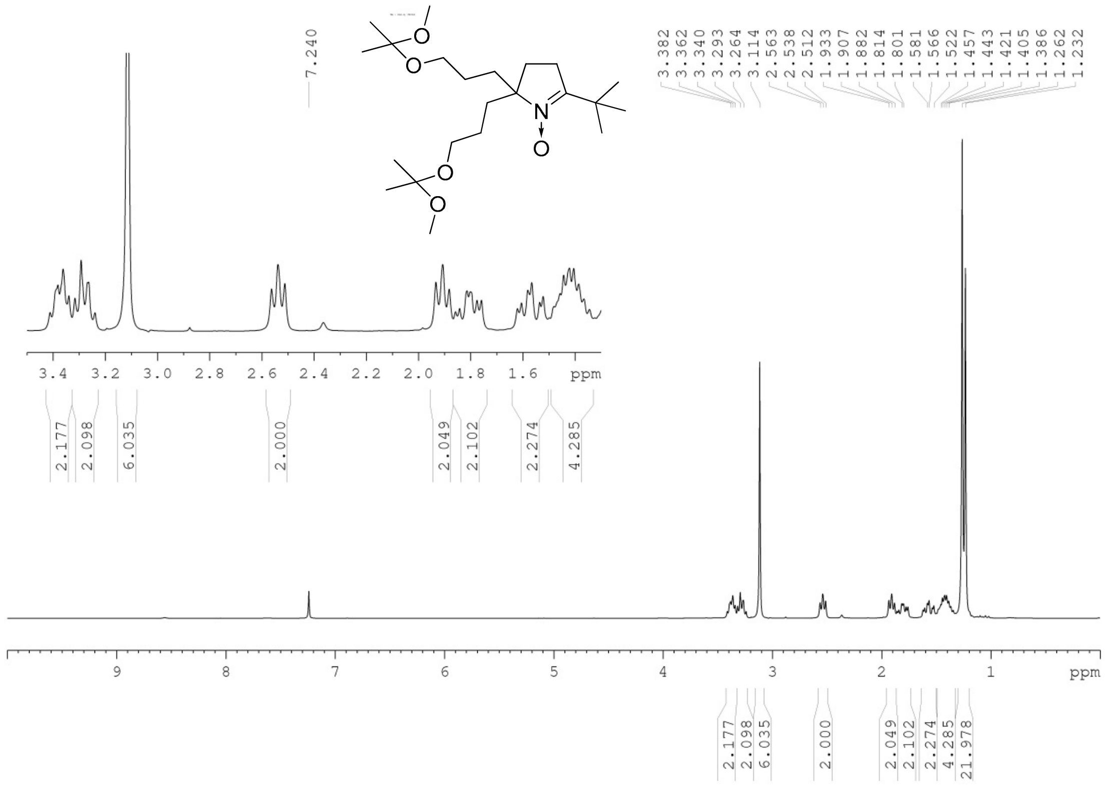

Figure S36. ^{1}H NMR spectrum of **12b** in $CDCl_3$ at 300 MHz

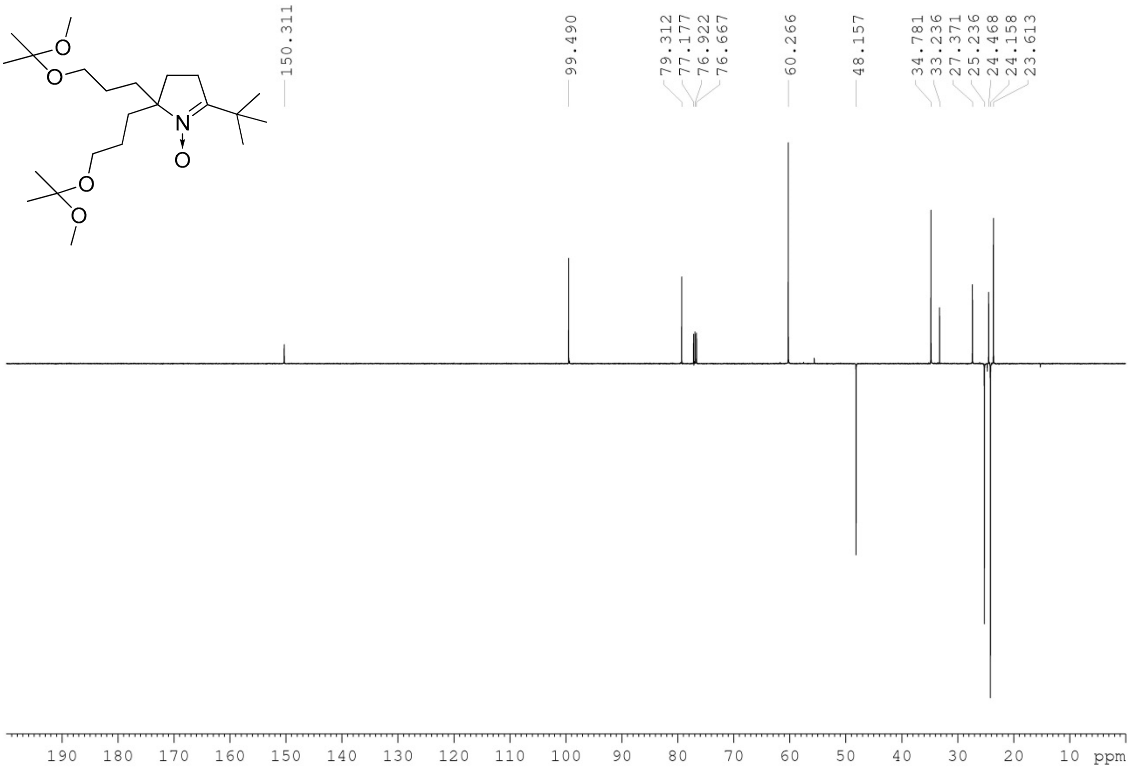

Figure S37. ^{13}C NMR spectrum of **12b** in $CDCl_3$ at 125 MHz

2.12 2-*tert*-Butyl-2,5-diethyl-5-(3-hydroxypropyl)pyrrolidinium trifluoroacetate (mixture of daistereomers) (**14a_*red***)

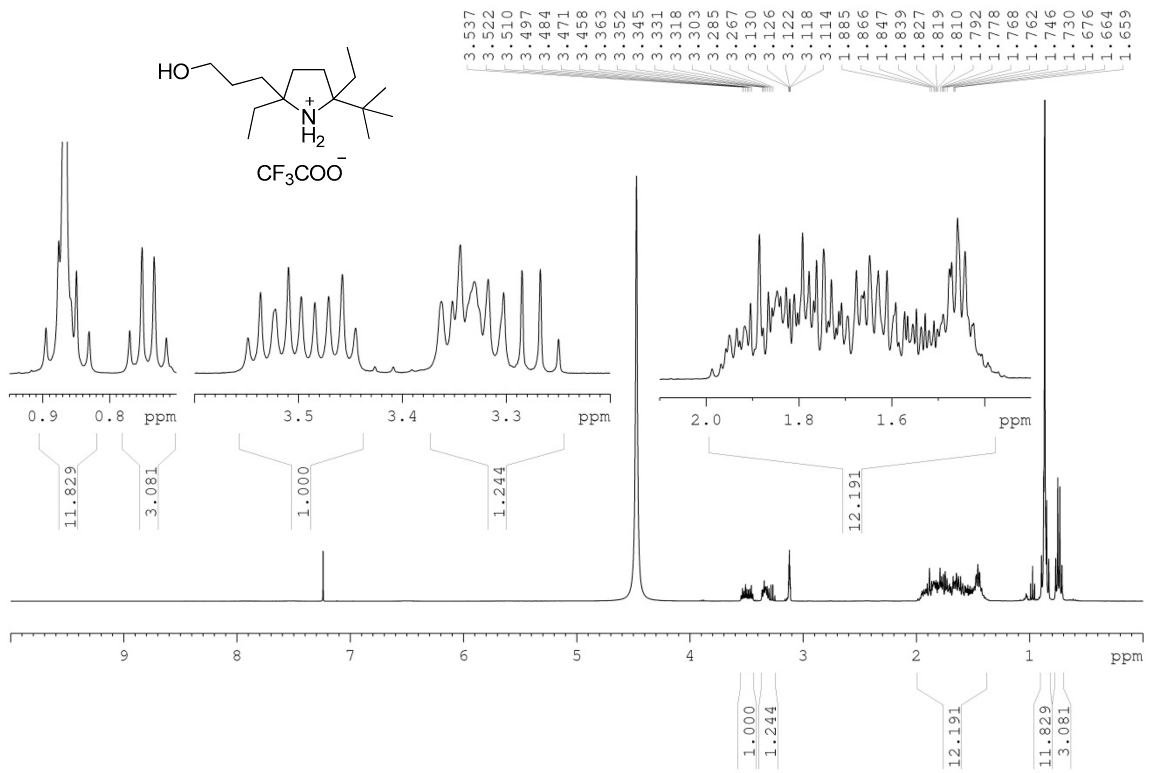

Figure S38. ^{1}H NMR spectrum of **14a_*red*** in $CDCl_3$-CD_3OD at 400 MHz

2.13 2-*tert*-Butyl-2-ethyl-5,5-bis(3-hydroxypropyl)pyrrolidinium trifluoroacetate (**14b_*red***)

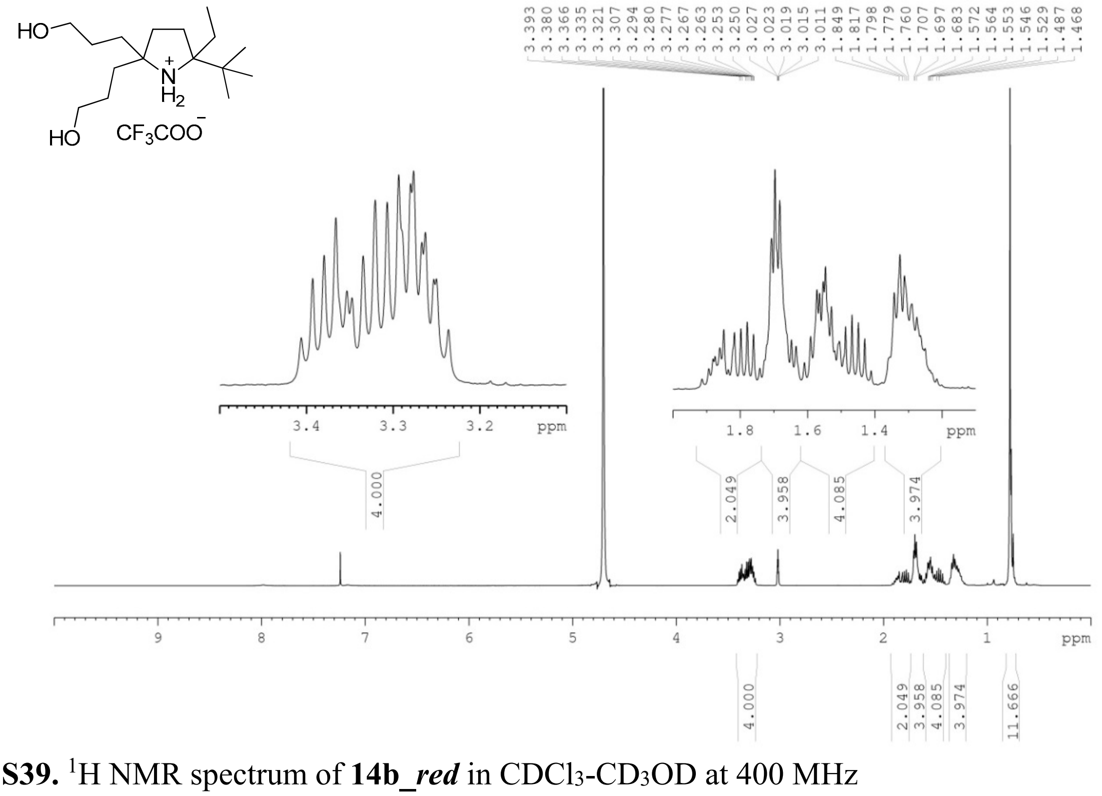

Figure S39. ^{1}H NMR spectrum of **14b_*red*** in $CDCl_3$-CD_3OD at 400 MHz

3. HPLC analysis

3.1 HPLC analysis of 2-*tert*-Butyl-2-ethyl-5,5-bis(3-hydroxypropyl)pyrrolidine-1-oxyl (**14a**)

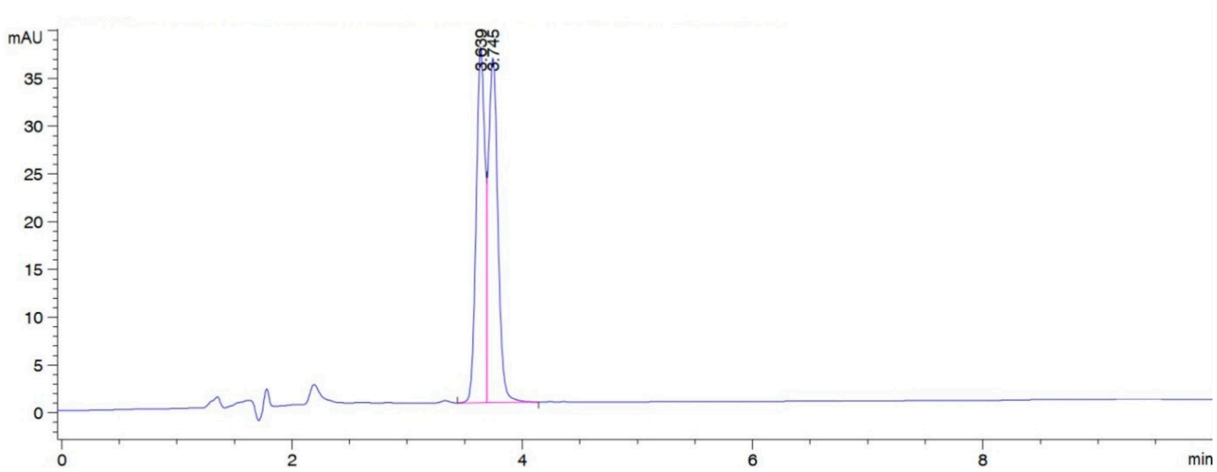

Figure S40. The HPLC analysis of **14a**
